# Testing the construct validity of competing measurement approaches to probed mind-wandering reports

**DOI:** 10.3758/s13428-021-01557-x

**Published:** 2021-04-09

**Authors:** Michael J. Kane, Bridget A. Smeekens, Matt E. Meier, Matthew S. Welhaf, Natalie E. Phillips

**Affiliations:** 1 Department of Psychology, University of North Carolina at Greensboro, P.O. Box 26170, Greensboro, NC 27402-6170 USA; 2 Western Carolina University, Cullowhee, NC 28723 USA

**Keywords:** Mind-wandering, Consciousness, Experience sampling, Measurement, Construct validity

## Abstract

Psychology faces a measurement crisis, and mind-wandering research is not immune. The present study explored the construct validity of probed mind-wandering reports (i.e., reports of task-unrelated thought [TUT]) with a combined experimental and individual-differences approach. We examined laboratory data from over 1000 undergraduates at two U.S. institutions, who responded to one of four different thought-probe types across two cognitive tasks. We asked a fundamental measurement question: Do different probe types yield different results, either in terms of average reports (average TUT rates, TUT-report confidence ratings), or in terms of TUT-report *associations*, such as TUT rate or confidence stability across tasks, or between TUT reports and other consciousness-related constructs (retrospective mind-wandering ratings, executive-control performance, and broad questionnaire trait assessments of distractibility–restlessness and positive-constructive daydreaming)? Our primary analyses compared probes that asked subjects to report on different dimensions of experience: TUT-content probes asked about *what* they’d been mind-wandering about, TUT-intentionality probes asked about *why* they were mind-wandering, and TUT-depth probes asked about the *extent* (on a rating scale) of their mind-wandering. Our secondary analyses compared thought-content probes that did versus didn’t offer an option to report performance-evaluative thoughts. Our findings provide some “good news”—that some mind-wandering findings are robust across probing methods—and some “bad news”—that some findings are not robust across methods and that some commonly used probing methods may not tell us what we think they do. Our results lead us to provisionally recommend content-report probes rather than intentionality- or depth-report probes for most mind-wandering research.

**Supplementary Information:**

The online version contains supplementary material available at 10.3758/s13428-021-01557-x.

A common view is that any study finding an effect under noisy conditions provides evidence that the underlying effect is particularly strong and robust. Yet, statistical significance conveys very little information when measurements are noisy. In noisy research settings, poor measurement can contribute to exaggerated estimates of effect size. This problem and related misunderstandings are key components in a feedback loop that perpetuates the replication crisis in science (Loken & Gelman, 2017, p. 584).

…[P]erhaps, there are even some who are of the opinion that things are actually going rather well in psychological measurement. This is not the case. The daily practice of psychological measurement is plagued by highly questionable interpretations of psychological test scores, which are directly related to the lack of integration between psychometrics and psychology. (Borsboom, 2006, p. 426)

Studies of mind-wandering, therefore, highlight one of the fundamental paradoxes in studying conscious experience: without the capacity for metacognitive access to our experiences, studies of conscious experience would be almost impossible; however, our access to our own experience means that the method of inquiry as part of an experiment may fundamentally alter the conscious experience itself (Konishi & Smallwood, 2016, p. 5)

Psychology is striving to improve its methodological practices to increase the information value of its empirical literature and the soundness of its theory. Journals and other institutions (such as open-science archiving platforms and granting agencies) now support well-powered designs, preregistration of hypotheses and analysis plans, alternative statistical approaches, replication of important findings, and open sharing of code and data. Yet these solutions to questionable research practices deal only indirectly with questionable *measurement* practices that impede scientific progress (e.g., Borsboom, 2006). Meta-scientific research on psychology, particularly in domains using self-reports, highlights a history of such poor measurement that Flake and Fried (2020) suggest we are “plagued by a *measurement schmeasurement* attitude.”

Most self-report measures in counseling psychology, for example, were reported without any psychometric properties and the modal number of items per scale was one (Meier & Davis, 1990). In seven health journals (2007–2010), 40–93% of scales were reported with no validation evidence and 35–80% with no reliability statistics (Barry et al., 2014). Articles published on psychological scales in *Emotion*, from 2001–2011, mostly described non-validated, one-item measures of unknown reliability (Weidman et al., 2017). In the *Journal of Personality and Social Psychology*, 80% of self-report scales published in 2014 included reliability data, but many reliabilities were inadequate, and most articles provided no additional psychometric information (Flake et al., 2017). Even more concerning than weak reporting practices is that validity information may be missing *systematically* from the literature. Hussey and Hughes (2020) analyzed a large dataset including 15 published psychological scales (*n*s ≈ 6700 per scale) and found that scales with less published validity evidence showed poorer psychometric properties. Many self-report instruments in psychology appear to be of poor or unknown quality.

## Measurement of mind wandering

In 2006, Smallwood and Schooler published their seminal review of the emerging scientific literature on mind wandering. The next decade and a half saw rapid growth in mind-wandering research, particularly within cognitive psychology and neuroscience (see Callard et al., 2013), but also across such diverse contexts as aeronautics and astronautics (e.g., Casner & Schooler, 2014; Gontier, 2017), education (e.g., Wammes, Boucher, et al., 2016; Wammes, Seli, et al., 2016), human factors (e.g., Burdett et al., 2019; Walker & Trick, 2018), lifespan development (e.g., Jackson & Balota, 2012; Soemer et al., 2019; Stawarczyk et al., 2014), personality (e.g., Perkins et al., 2015; Robison et al., 2017), philosophy (e.g., Irving, 2016; Metzinger, 2013), and psychopathology (e.g., Chen et al., 2021; Hoffmann et al., 2018; Lanier et al., 2021; Makovac et al., 2019). And, despite a research pace and impact that has supported numerous reviews and theoretical commentaries (e.g., Christoff & Fox, 2018; Klinger, 2013; Mildner & Tamir, 2019; Mittner et al., 2016; Smallwood & Andrews-Hanna, 2013; Smallwood & Schooler, 2015), little published work has focused on the validity of subjects’ self-reported mind-wandering experiences. This gap is surprising, given psychologists’ general caution regarding introspective methods. In 2018, however, Head and Helton published a critique of mind-wandering research practices of probing subjects’ thought content within tasks. Weinstein (2018) followed with a review identifying nearly 70 ways in which mind wandering had been assessed in thought-probe studies, all without considering potential consequences for valid measurement. These critiques reminded mind-wandering researchers of the potential perils of self-reports and the value of skeptically validating introspective data.

Psychological studies assess mind wandering in numerous ways that may vary in validity. Subjects might complete retrospective questionnaires about their mind-wandering frequency, or they might signal every time they realize they are mind-wandering during a task. The most common method, however, engages subjects in an activity that is unpredictably interrupted with thought probes to classify their immediately preceding thoughts (as an open-ended question, a continuous rating scale, or a forced choice among options). Such probed reports of task-unrelated thoughts (TUTs) should provide higher fidelity reports than other methods because they don’t require retrospection about ephemeral experiences (and aggregating them over minutes, hours, or weeks), and they don’t ask subjects to continuously monitor their subjective experiences.^1^ The present study assesses the validity of such minimally retrospective, probed TUT reports.

## General concerns about introspective self-reports

Psychological scientists may be among the most reliant on—and skeptical of—self-report data (Haeffel & Howard, 2010). That skepticism is long-standing, commonly understood to arise from the demise of Titchener’s structuralism (founded upon “systematic introspection”) and the ascendance of Watson’s behaviorism in the early 20^th^ century.

### Introspection and the imageless-thought controversy

As undergraduates learn in introductory courses (e.g., Anderson, 2005; Jahnke & Nowaczyk, 1998; Reisberg, 2016), early disagreements across laboratories about the existence of imageless thought could not be resolved from subjects’ first-person reports, thus leading the fledgling scientific field to abandon introspection as a central method. The problem with this narrative (see Hurlburt & Heavey, 2001), is that disagreements about imageless thought were not in the self-reports, but rather in theorists’ *explanations* for them. Monson and Hurlburt (1993) revisited the original investigations and found that it wasn’t only the subjects tested by the Külpe-Würzburg (pro-imageless-thought) school who described non-sensory, non-symbolic thoughts. Subjects tested in the anti-imageless-thought laboratories did, too, but their reports were explained away by the investigators. It was the theorists—*not the subjects*—who disagreed. Using modern methods, Hurlburt and colleagues regularly obtain reports of imageless, unsymbolized thinking (e.g., Heavey & Hurlburt, 2008; Hurlburt & Akhter, 2008). Irresolvable disagreements about introspections are thus not a likely barrier to valid TUT reports.

### Faulty introspections biased by implicit causal theories

Hurlburt and Heavey (2001) noted a second concern about introspection: Self-reports are frequently biased and demonstrably incorrect (Nisbett & Wilson, 1977). People misreport reasons for their behaviors and experiences because: (a) many mental processes are consciously unavailable, yet (b) we have implicit theories to draw upon for “explanation” (see also Haidt et al., 2000; T. D. Wilson & Stone, 1985). These introspective errors are especially likely when subtle, surprising, or temporally distant events influence behavior.

Many skeptics of introspection forget, however, that Nisbett and Wilson (1977) distinguished the frequently erroneous self-reports about causal cognitive processes from those about the contents of consciousness (see also T. D. Wilson, 1994, 2002):

The individual…knows the focus of his attention at any given point in time; he knows what his current sensations are and has what almost all psychologists and philosophers would assert to be “knowledge” at least quantitatively superior to that of observers concerning his emotions, evaluations, and plans. (Nisbett & Wilson, 1977, p. 255)

Although people frequently err about the *reasons why* they feel, think, or do something, this does not imply that they frequently err about *what* they feel, think, or do. If thought reports focus on *what* is experienced rather than on *why* the experience came to be, then they should avoid biases from implicit causal theories. But introspective reports about consciousness can go awry in many ways, so obtaining maximally valid self-reports of mind-wandering is not trivial.

## Descriptive experience sampling and careful reflections on valid self-reports

Hurlburt (1990, 1993, 2011) developed “Descriptive Experience Sampling” (DES) to study consciousness in daily life. Subjects wear an earpiece that beeps unpredictably; at each beep, subjects take stock (and written notes) of their momentary experience. Later, subjects engage in a collaborative interview with the investigator about each beep to clarify the experience. Perhaps because most DES reports can’t be corroborated by objective evidence (but see Hurlburt, 1993; Kühn et al., 2014), DES’s development has focused intensely on optimizing validity. Following Nisbett and Wilson (1977), subjects report experiences but not inferences about them to avoid bias from folk theories. Subjects report only experiences immediately preceding the signal to minimize forgetting and confabulation. To limit influence of implicit theories, subjects report on randomly selected episodes rather than on generalities or self-selected contexts. The introspection signal (i.e., beep) is clear and unambiguous to prevent interference from extraneous thoughts. DES minimizes demand characteristics by encouraging subjects to report all kinds of subjective experiences, to not lead the witness. Finally, the investigator and subject collaborate to clarify the subject’s vocabulary for describing experiences; subjects iteratively learn to communicate these experiences via feedback.

Whether or not DES is a “gold standard” self-report method, it is instructive to compare other methods to one that has taken validity so seriously. In fact, most studies that assess TUTs with thought probes share many DES features: Subjects typically report what they were thinking in the instant before the probe, and clearly signaled probes appear unpredictably. But some mind-wandering studies don’t meet these criteria, and most fall short of other DES principles. Let’s briefly consider three examples:

- Killingsworth and Gilbert (2010) probed over 2000 subjects for TUTs in daily life via a smartphone app. People receive many signals from their phones (e.g., calls, texts, notifications), so subjects likely didn’t always attend to their fleeting thought content at the signal. Moreover, subjects responded to a happiness question before reporting thought content, allowing further forgetting and, perhaps, reactivity to the mood question. Together, these design choices may have caused the higher-than-typical TUT rate found in this study (~50% vs. ~25–35%; Franklin, Mrazek, et al., 2013; Kane et al., 2007, Kane, Gross et al., 2017; Marcusson-Clavertz et al., 2016; McVay et al., 2009; Seli, Beaty et al., 2018; Song & Wang, 2012).
- Seli, Smilek, and colleagues have distinguished intentional and unintentional mind wandering (e.g., Seli et al., 2015; Seli, Risko, & Smilek, 2016a, 2016b), which is potentially critical for theory and practice (but see Murray and Krasich 2021). Probes ask whether thoughts were on-task, intentionally off-task, or unintentionally off-task. The potential concern is that subjects must convey not only the “what” of experience (i.e., whether thoughts were about the task), but also the “why” (i.e., whether mind-wandering was deliberate). Subjects’ reports might be influenced by implicit causal theories or by forgetting and confabulating how a TUT episode began or was maintained.
- Christoff, Irving, and colleagues argue that unconstrained thought movement is the defining feature of mind wandering (Christoff et al., 2016; Irving, 2016), and so Mills et al. (2018) asked subjects to report at each probe not only whether their thoughts were off-task but also whether they were freely moving. Although they instructed subjects to take a “mental snapshot” of the instant of the probe, thought movement cannot be determined from an instant, it must be monitored then recalled over time. Thus, probes for unconstrained, freely moving thought require retrospection over seconds, or minutes, and subjects may vary in how far back and how accurately they retrospect.

Although none of these examples is certain to reflect low fidelity self-reports, methods like these should prompt a skeptical consideration of validity.

Moreover, *most* mind-wandering studies using probed self-reports fail other DES criteria. They don’t consider demand characteristics of focusing subjects’ reports on, and repeatedly asking about, TUTs. Few studies describe how their instructions defined mind wandering, and so subjects may interpret the term or its connotations differently, biasing their reporting (see Seli, Kane, Smallwood, et al., 2018). Most investigators assume subjects comply with instructions to report only on immediately preceding thoughts, but Hurlburt and Heavey (2015) observe that many DES subjects only do so after iterative practice. Notably, few studies provide any thought-probe practice (Hu et al., 2012; Kane et al., 2016). Given the potential for bias and error, psychologists should critically examine the evidence for thought-report validity.

## Evidence for probed TUT report validity

Construct validation involves the building and testing of a nomological net and the specification of causal processes for experiences and behaviors via inference from empirical findings (e.g., Borsboom et al., 2004; Cronbach & Meehl, 1955; Embretson, 1983). Researchers can thus find evidence in the mind-wandering literature that probed thought reports are valid. Even studies that were not conducted to assess the validity of TUT reports can inform whether they capture the construct.

We first consider measurement reliability as a condition for validity. In fact, individual differences in TUT rates are reliable across task and temporal contexts. Latent-variable studies that probe thoughts in multiple tasks across occasions find that TUT rates elicit a unitary factor (e.g., Hollis & Was, 2016; Kane et al., 2016; McVay & Kane, 2012b; Robison & Unsworth, 2017, 2018; Unsworth & McMillan, 2014; Unsworth & Robison, 2017b). People who mind-wander more in one task (and lab session) tend to mind-wander more in other tasks (and sessions), although TUT rates within laboratory tasks correlate only modestly-to-weakly with those from daily-life experience sampling (Kane, Gross et al. 2017; McVay et al., 2009). Measurable reliability of probed TUT rates does not guarantee the validity of thought reports, of course, because reliability might partially reflect consistency in implicit theories, (dis)honesty, or reactivity to task performance.

Substantive validity evidence comes from TUT rates varying in predictable ways with experimental manipulations of, and natural variation in, the activity context. Subjects report fewer TUTs during difficult than easy tasks (e.g., Rummel & Boywitt, 2014; Teasdale et al., 1993), during faster- than slower-paced tasks (e.g., Antrobus, 1968; Giambra, 1995), during less- than more-practiced tasks (e.g., Mason et al., 2007; Teasdale et al., 1995), and during earlier than later trials within a task (e.g., Antrobus et al., 1967; McVay & Kane, 2009; Thomson et al., 2014), even when some of these are manipulated between subjects, effectively blinding subjects to comparison of interest. TUTs also predict performance: Task error rates are higher, and RTs are more variable, immediately preceding TUT than on-task reports (e.g., Bastian & Sakur, 2013; McVay & Kane, 2012a; Seli, Cheyne, & Smilek, 2013; Smallwood et al., 2007; Wammes, Seli, et al., 2016), and subjects who report more TUTs also tend to perform worse (e.g., McVay & Kane, 2012b; Wammes, Seli, et al., 2016).

Of importance to validity, TUT–performance associations arise even: (a) for non-introspectable aspects of performance, such as intrasubject RT variability (e.g., Bastian & Sakur, 2013; Kam et al., 2012; McVay & Kane, 2012a); (b) in tasks without overt cues to performance, such as reading and lecture comprehension (e.g., Hollis & Was, 2016; Smallwood et al., 2008) and implicit learning (Franklin et al., 2016), and; (c) following errors that subjects have not detected and for subjects who detect few errors (Allen et al., 2013). Thus, TUT reports predict overt markers of inattention, even without performance feedback.

Finally, TUT reports covary with external indicators that are independent of subjects’ folk theories or performance reactivity. Psychophysiological and neuroscience methods demonstrate associations between TUT reports and pupil dilation (e.g., Franklin, Broadway, et al., 2013; Konishi et al., 2017; Unsworth & Robison, 2016), EEGs (e.g., Baird et al., 2014; Compton et al., 2019; Kam et al., 2011), and default-network activity (e.g., Christoff et al., 2009; Kucyi, 2018; Mittner et al., 2016). Ability constructs, such as working memory capacity and intelligence, predict TUT rates in lab tasks and daily-life contexts (e.g., Kane & McVay, 2012; Mrazek et al., 2012; Unsworth & Robison, 2017a). Subjects cannot intuit their neurophysiological responses, and they don’t know their standing on many cognitive constructs (and don’t have folk-theoretical commitments about their relations to TUTs), thus supporting the construct validity of TUT reports.

Together, these findings suggest that probed TUT reports are valid measures of mind wandering. We limited our discussion to results that were relatively immune to subjects’ beliefs, causal theories, or reactions to performance, thus providing compelling tests of construct validity. These studies, however, were not designed to skeptically evaluate validity and they didn’t compare probing methods to maximize validity or assess relative validity. We thus turn to recent research designed to interrogate the validity of TUT reports.

## Explicit validation studies and concerns about probed mind-wandering reports

Few studies have rigorously evaluated the construct validity of probed thought reports. We first consider whether probes might alter subjects’ experiences or reports (Konishi & Smallwood, 2016). Next, we evaluate whether TUT reports may be contaminated by subjects’ awareness of task accuracy, explaining errors by inferring TUTs (Head & Helton, 2018). We then review the potentially biasing effects of different probe framings (Weinstein, 2018). Finally, we assess whether demand characteristics influence probed TUT reports.

### Reactivity to probing

The reactivity of TUT reports to probing was assessed by four studies. Three tested whether probe rate affected TUT rates: Robison et al. (2019) probed subjects after 7% or 13% of task trials, Schubert et al. (2020) probed after 3% or 6% of trials, and Seli, Carriere, et al. (2013) presented 5–25 probes in a 15-min task . Results varied. Robison et al. (2019) found no probing effect on TUT rates, but both other studies found that higher probe rates yielded lower TUT rates. Regarding individual differences, Schubert et al. (2020) found no interactions of probe rate with theoretically informed covariates (such as working memory capacity) in predicting TUT rate. Varao-Sousa and Kingstone (2019) assessed whether students’ rate of *self-caught* TUTs during three lectures varied by including probes in only one of them. Self-caught TUTs (and their correlations with motivation and interest) did not differ across lectures. Thus, the few relevant studies provide inconsistent evidence that probe rate can alter reports, and none show probe rate to affect individual differences in TUTs.

### Reactivity to performance

Head and Helton (2018) presented a go/no-go “SART” task with digits as the imperative stimuli; subjects in different between-subject conditions also saw incidental words or control screens between the digits, including prior to the final “catch trial” of the task. After the catch trial, subjects were asked whether they had just seen a word, then tried to recognize it among foils, and then reported their preceding thought content. Catch-trial TUT reports varied with catch-trial go/no-go accuracy, but not with memory performance. The authors thus argued that when probes follow no-go trials that frequently produce errors, performance appraisals bias subjects to report TUTs. In contrast, when performance accuracy is not salient, such as noticing an incidental word, TUT reports are not affected by performance. Unfortunately, Head and Hilton’s catch-trial method wasn’t a fair test because it virtually guaranteed invalid self-reports: It inserted *multiple* unexpected memory questions between the experience and report—thus interfering with access to the targeted conscious state; moreover, subjects made only the *one* thought report in the entire task, without forewarning, and only after reading multiple sentences defining mind wandering following the catch-trial memory tasks (D. Helton, personal communication, April 9, 2016).

Taking a more straightforward approach, however, Schubert et al. (2020) found evidence for reactivity by presenting probes after no-go and go SART trials, the former of which elicit salient errors. Subjects reported more TUTs following no-go than go trials, and more TUTs following no-go errors than accurate no-go responses. Both effects suggest some reactivity to performance. At the same time, go/no-go trial type did not interact with most variables of theoretical interest to predict TUTs, such as working memory capacity. The Schubert et al. (2020) findings thus indicate that TUT reports can be somewhat reactive to errors, affecting mean TUT rates, but they don’t convincingly demonstrate that mind wandering’s associations with other constructs reflect performance reactivity.^2^

### Biases from thought-probe framing

Four studies addressed Weinstein’s (2018) concern that the blithe proliferation of probing methods, without careful validation or consultation of self-report research (e.g., Krosnick, 1999; Schwarz, 1999), may hinder cumulative science. In two separate experiments, Robison et al. (2019) examined whether the instructional framing of mind-wandering as more positive or negative, or whether the number of thought-report options in each probe, would affect TUT reports. Instructional framing had no measurable effects. However, when probes offered only two response options (on-task vs. TUT), TUT rates were higher than with three response options (including “off-task” thoughts about one’s performance), and these were higher than with five options (including externally-oriented distractions and mind-blanking). Thus, subjects’ classification of TUTs may depend to some degree on the response options available.

Both Weinstein et al. (2018) and Schubert et al. (2020) tested whether subtle differences in probe framing influence TUT rates: Probes asked subjects whether they had just been mind-wandering (yes/no), or whether they had just been on-task (yes/no) in Weinstein et al. (2018), and probes presented the response options “on-task” on the left and “off-task” on the right—or vice versa—in Schubert et al. (2020). In Weinstein et al. (2018), mind-wandering-framed probes elicited higher TUT rates (M ± SD = 34 ± 23%) than did on-task-framed probes (23 ± 21%). In contrast, in Schubert et al. (2020), left–right framed probes had no effect on TUTs and no interactions with performance, probe rate, or working memory (a few higher-order, unpredicted interactions involving probe framing should be replicated before conclusions are warranted). In summary, probe framing and probe response options may have some modest effects on mean TUT rates, but we don’t yet have evidence for their effects in relation to experimental manipulations or individual differences.

### Biases from demand characteristics

Two studies tested for demand characteristics. Zedelius et al. (2015) compared a control group to those told that an eyetracker would monitor their eyes while reading to verify their TUT reports. Probed TUT rates didn’t differ among groups. In contrast, Vinski and Watter (2012) primed honesty in half their subjects with a synonym task (Rasinski et al., 2005). Mean TUT rate during a subsequent SART was higher for the control than the honesty group, suggesting that controls over-reported TUTs. However, only the honesty group showed a prototypical RT effect, with faster go-trial RTs preceding TUTs than on task-reports, and it’s unclear why the control group did not. Given this odd RT pattern and the fact that, like many goal-priming studies, the original Rasinski honesty-prime finding has failed direct replication attempts (Pashler et al., 2013), we are skeptical of the findings. We see little compelling evidence for demand effects on TUT reports.

## The present study: Goals, questions, and approach

Mind-wandering research has largely assumed that different methods yield comparably valid results (Weinstein, 2018). Recent studies of probe methods, however, suggest validity threats: TUT rates may be influenced by probing rate, reactivity to errors, and probe framing. The present study further assessed the construct validity of TUT reports. The primary aim was to test whether TUT-rate correlations, as well as mean TUT rates, were affected by probing subjects for three different report types used frequently in the literature.

Although it is of limited interest that probe types affect *mean* TUT rates (Robison et al., 2019; Weinstein et al., 2018), theoretical claims about mind wandering hinge on *variation* in TUT rates in response to experimental manipulations or in association with individual-differences constructs. Only Schubert et al. (2020) tested competing probes by correlating TUT rates with other variables. The present study further does so by probing in two tasks and by testing correlations with numerous theoretically motivated variables.

In contrast to subtle probe variations investigated previously (Robison et al., 2019; Schubert et al., 2020; Weinstein et al., 2018), which may underestimate probe-framing effects, the present study’s three primary probe types asked for reports about distinct thought dimensions: (a) TUT content, or the “*what*” of subjects’ experience (thinking about the task versus thinking about one’s current state, worries, everyday things, external distractions, or daydreams; e.g., McVay & Kane, 2009; Smallwood et al., 2011); (b) TUT intentionality, or the “*why*” of subjects’ experience (thinking about the task versus intentionally or unintentionally mind-wandering; e.g., Forster & Lavie, 2009; Seli, Risko, & Smilek (2016a); or, (c) TUT depth, or the “*how much*” of subjects’ experience (the graded extent to which their thoughts were on-task versus off-task; Christoff et al., 2009; Franklin et al., 2011; Mrazek et al., 2012). Each of these distinct probe types has been used frequently in the literature and so our conclusions should apply directly to prior (and future) mind-wandering research. Our main analytic approach collapsed over all response options reflecting TUTs in each probe-type and then compared TUT rates and TUT-rate associations across methods. Subsequent fine-grained analyses explored whether TUT-intentionality and TUT-depth reports were vulnerable to validity threats commonly associated with self-reports of intention (e.g., Nisbett & Wilson, 1977) and numerical rating scales (e.g., DuBois & Burns, 1975; Schwarz, 1999).

Our secondary aim was to explore the consequences of thought probes not including an option to report evaluative thoughts about performance (“task-related interference” [TRI]; Matthews et al., 1999; Smallwood, Obansawin, & Heim 2003). Subjects report TRI in response to both closed-ended (e.g., Jordano & Touron, 2017; Kane, Smeekens, et al., 2017; Mrazek et al., 2011; Stawarczyk et al., 2011) and open-ended probes (Jordano, 2018). Most studies of TUT, however, do *not* include a TRI response option, so TRI must be reported as another category (perhaps as on-task, as it’s task-*related*). Robison et al. (2019) used probes that included a TRI option or not. Although TUT rates changed somewhat across probe groups, on-task reports changed dramatically, suggesting that most TRI reports in no-TRI conditions are reported as on-task. The present study conceptually replicates and extends Robison et al.

To address all study aims about TUT measurement, we tested a large subject sample from two U.S. universities in two tasks with embedded thought probes, manipulating probe types between subjects (*n*s > 260 per group), following each thought probe with a confidence rating prompt, and following each probed task with a retrospective questionnaire assessment of off-task thinking. Across probe-type conditions, our analyses will compare TUT rate means and consistency across probed tasks, TUT-report confidence, associations with in-the-moment RT variability, associations with in-the-moment response accuracy, correlations with retrospective off-task reports, correlations with a composite measure of executive-control performance, correlations with a composite questionnaire measure of distractibility and restlessness, and correlations with a composite questionnaire measure of positive-constructive daydreaming. Thus, the study primarily takes a combined experimental–individual-differences approach to rigorously assess the construct validity of probed TUT rates (and TRI rates) elicited by different thought probes.

## Method

Below we report how we determined our sample size and all data exclusions, manipulations, and measures in the study (Simmons et al., 2012). IRBs at the University of North Carolina at Greensboro (UNCG) and Western Carolina University (WCU) approved the study.

### Subjects

We tested 760 UNCG and 348 WCU students (total *N* = 1108), from 2015 to 2018. UNCG and WCU are comprehensive state universities in North Carolina, USA, with UNCG in a more urban and WCU in a more rural setting; UNCG is a Minority-Serving Institution for African American students. Eligible subjects were 18–35 years old. We provide demographics for subjects from whom we analyzed data under *Results*.

Our stopping rule was the end of the semester in which we reached 210 subjects in each of four probe conditions who had completed the cognitive and questionnaire measures (exceeding the sample size to detect correlations ≥ .20 with two-tailed tests, an α-level of .05, and 80% power). We reached this mark early in the final semester and continued testing at UNCG to approximate 250 subjects per condition, allowing more precise correlation effect-size estimates in the .10–.20 range (Schönbrodt & Perugini, 2013). During the first two semesters at WCU (*N* = 173), subjects completed only the cognitive measures.

### Apparatus and materials

We programmed all measures in E-Prime 1.2 or 2.0 software (Psychology Software Tools, Pittsburgh, PA 2012). Mac Mini computers with Acer V226WL 22″ widescreen LCD monitors (at UNCG), and Dell OptiPlex 9020 minitower computers with Dell P2214H 22″ widescreen LCD monitors (at WCU) presented all stimuli and recorded all responses.

### Measures

We describe the cognitive tasks and self-report questionnaires in the order in which subjects completed them, followed by descriptions of the thought probes that appeared within two tasks.

#### Antisaccade letters (ANTI-LET)

On each of 90 trials, subjects directed attention away from a salient flashing cue to identify a masked letter (*B, P*, or *R*) at opposite side of the screen. Trials began with a central fixation array (***) for 200–1800 ms (in 400 ms increments). A flashing cue (=) then appeared 8.6 cm to the left or right for 100 ms, disappeared for 50 ms, appeared for 100 ms, then disappeared for 50 ms. The target letter then appeared 8.6 cm from fixation in the cue-opposite direction for 100 ms before being masked by an “H” (50 ms) then “8” (until response, or a maximum of 10 s). All stimuli appeared in Courier New 12 pt font. Subjects identified letters using keys labeled *B*, *P*, or *R* by stickers; a 400-ms blank screen followed each response. The task began with 36 trials of practice (12 trials for each letter) with masked target letters presented at central fixation, and then 12 practice trials with antisaccade cuing, all with visual accuracy feedback after each trial. Accuracy rate was the dependent variable. Due to a programming error, stimuli were presented slightly differently between the two sites. At WCU, the flashing cues and target arrows appeared 7.1 cm from fixation instead of 8.6 cm.

#### Semantic Sustained Attention to Response Task (SART)

On each trial, subjects pressed the space bar (“go”) when they saw an animal name (89% of trials) and withheld response (“no-go”) when they saw a vegetable name (11%). Each word appeared at fixation for 300 ms and was masked by 12 *X*s for 1500 ms. The program divided trials into five seamless blocks of 135, each comprising three mini blocks that each presented 40 unique animals (“go” trials) and five unique vegetables (“no-go” trials). Probes appeared after three of the five no-go trials in each mini block, for 45 total.

Subjects first practiced 10 trials presenting boys’ names for “go” and girls’ names for “no-go;” the real task began with 10 unanalyzed buffer trials and then 675 critical trials. Dependent measures were a d′ accuracy score and intraindividual RT variability (i.e., the SD of each subject’s go-trial RTs); we subtracted each subject’s RTsd from the maximum RTsd value in the dataset, so higher scores meant better performance.

#### Dundee Stress State Questionnaire 1 (DSSQ1)

Immediately following the SART, subjects answered 12 questions (in random order) about their experiences, drawn from the Thinking Content subscale of the DSSQ (dropping items 2, 6, 10, and 12; Matthews et al., 1999). Each item asked about thought frequency of various topics during the SART; subjects responded by clicking their choice along a 1–5 scale labeled, “Never,” “Once,” “A Few Times,” “Often,” and “Very Often.” Each question remained onscreen until response. Subjects could skip a question by clicking a “Submit” icon without having clicked on a response choice. A small pop-up box then asked, “*Did you mean to skip this question?*” along with a “*Yes*” and a “*No*” option; if the subject clicked “*Yes*” the program moved to the next item, but if the subject clicked “*No*” the program re-presented the item. We separately coded six questions related to thoughts about task performance as a “TRI” subscale, and six questions related to TUTs as a “TUT” subscale. We used mean ratings for TRI and TUT items as dependent variables.

#### Antisaccade arrows (ANTI-ARO)

This antisaccade task presented 72 trials presenting masked target arrows pointing left, right, up, or down. Trials began with a central fixation array (***) for 250–2250 ms (in 500 ms increments). A flashing cue (=) then appeared 17.0 cm to the left or right of fixation for 80 ms, disappeared for 50 ms, appeared for 80 ms, then disappeared for 50 ms. The target then appeared 17.0 cm from fixation in the cue-opposite direction for 100 ms before being masked by a “+” (50 ms) and a “❖” symbol (until response, or a maximum of 10 s). Subjects responded with the 2, 4, 8, and 6 keys on the number keypad for down, left, up, and right arrows, respectively. Twenty practice trials (5 trials for each direction) presented masked arrows at fixation and with visual accuracy feedback after each trial. We used accuracy rate as the dependent variable.

Due to a programming error, stimuli were presented differently between the two sites. At WCU, the cues and arrows appeared only 14.4 cm from fixation. Because of this large difference between sites (which produced a 13% accuracy improvement from ANTI-LET to ANTI-ARO for WCU subjects but a 1% drop for UNCG subjects), we z-scored accuracy from this task separately by site before combining data across sites.

#### Arrow flanker (FLANKER)

We used this task only as a secondary source of thought-probe data. On each of 192 trials (divided into two seamless blocks of 96), subjects reported the direction of an arrow at fixation (“<” vs. “>”) flanked by four distractors. After a 500 ms blank screen, a fixation cross (“+”) appeared for 350 ms, followed by the stimulus array, which presented either neutral flankers (“•”; 48 trials), congruent flankers pointing the same direction as the target (48 trials), incongruent flankers pointing the opposite direction as the target (48 trials), or incongruent flankers pointing upward (48 trials), until response. Subjects pressed the “z” key (labeled with an “L” sticker) for left-pointing targets and the “/” key (labeled with an “R” sticker) for right-pointing targets.

The task began with 10 practice trials without flankers, then 10 practice trials with flankers. Thought probes appeared after four of the first 96 test trials and after 16 of the second 96 trials, for 20 total; half the probes followed trials presenting incongruent flankers pointing the opposite direction of the target and half followed trials presenting flankers pointing upwards.

#### Dundee Stress State Questionnaire 2 (DSSQ2)

Immediately following the flanker task, subjects again completed the DSSQ, here about thoughts during the flanker task. We analyzed mean ratings for the six TRI items and for the six TUT items.

#### Questionnaire battery

Following the cognitive tasks, subjects completed a 202-item self-report battery made up of items from several scales. Items appeared in a random order for each subject, except for 20 items at fixed positions to assess careless responding. All items presented a 1–5 response scale, labeled from left to right as, “Strongly Disagree,” “Disagree,” “Neutral,” “Agree,” and “Strongly Agree,” although not all the original questionnaires used this response format. As with the DSSQ, subjects could choose to skip individual questions and were prompted with a confirmation pop-up if they did so. We first describe the scales of interest from which we drew items (in alphabetical order), and then the scales that assessed inattentive and careless responding. For all scales of interest, we computed a mean score after reverse-scoring appropriate items.

##### AD/HD Rating Scale IV–Self-Report Version

We included the first 18 of 20 items (DuPaul et al., 1998), none of which explicitly mentioned AD/HD; nine items asked about inattentiveness symptoms (e.g., making careless mistakes) and 9 about hyperactivity (e.g., talking excessively). We modified all items to ask about childhood symptoms by beginning them with either, “*During childhood…*,” “*As a child…*,” or “*When I was young…*” We derived separate inattentiveness and hyperactivity scores from the scale.

##### Cognitive Failures Questionnaire–Memory and Attention Lapses (CFQ–MAL)

Based on principal components analysis from McVay and Kane (2009), we selected the top 12 loading items (all > .60) not mentioning mind wandering or daydreaming. Items asked about failures such as forgetting things at home and leaving a step out of a task (two questions were *related* to mind wandering: keeping one’s mind on a job and failing to notice one hasn’t been attending to an ongoing activity). Because the original subscale used frequencies (e.g., “never”, “very often”) as response options, we revised some items to fit a disagree–agree scale (e.g., we revised “*Are you unable to find something that you put away only a couple of days ago?*” to “*I’m often unable to find something that I put away only a couple of days ago.*”); these revisions yielded seven regularly scored items (higher ratings = more failures) and five reverse-scored items.

##### Creative Achievement Scale (CAS)

We included 12 of the original CAS items, about music, visual arts, or writing (four items each; Carson et al., 2005). In each domain, items asked about progressively more significant accomplishments. For example, in visual art, questions asked about taking art lessons, winning a prize at a juried art show, selling a piece of art, and having artwork critiqued in a significant publication.

##### Imaginal Process Inventory (IPI) Boredom scale

This IPI (Singer & Antrobus, 1970) subscale presented 12 items about being easily bored (with reverse-scored items about interest in everyday things).

##### IPI–Daydreaming Frequency scale

This IPI subscale presented 12 items about being lost in thought. Because the original subscale used frequencies as response options, we revised some items to fit a disagree–agree scale (e.g., we revised “*I daydream*” to “*I daydream frequently*”); these revisions yielded 10 regularly scored items (higher ratings = more daydreaming) and 2 reverse-scored items.

##### IPI–Mentation Rate scale

This IPI subscale presented 12 items about experiencing racing and active thoughts (with reverse-scored items about slow thoughts and mind-blanking).

##### IPI–Mind Wandering scale

This IPI subscale presented 12 items about mind-wandering frequency and concentration difficulties (with reverse-scored items about easily focusing).

##### IPI–Problem Solving Daydreams scale

This IPI subscale presented 12 items about solving problems, and seeing importance, in daydreams (with reverse-scored items about non-pragmatic daydreams).

##### Schizotypy–Magical Ideation scale

We used all 15 items from the short form of the Magical Ideation scale (Winterstein et al., 2011). This measure, designed to assess a dimension of positive schizotypy, asked about beliefs and experiences reflecting paranormal, superstitious, or bizarre influences on thought and behavior, or about everyday events having referential meaning (e.g., “*I have sometimes felt that strangers were reading my mind*;” “*I have occasionally had the silly feeling that a TV or radio broadcaster knew I was listening to him*.”); one reverse-scored item denied a common superstition.

##### Metacognitive Prospective Memory Battery

The questionnaire included only the seven “internal” items from the measure from Rummel et al. (2019), asking about strategies for fulfilling intentions not involving external aids (e.g., “*In my mind, I make a list of things that I still have to complete.*”).

##### Mind Wandering–Deliberate scale

The four items from Carriere et al. (2013) asked about intentional mind wandering (e.g., “*I find mind-wandering is a good way to cope with boredom*;” “*I allow my thoughts to wander on purpose*.”).

##### Mind Wandering–Spontaneous scale

The four items from Carriere et al. (2013) asked about propensity for unintentional mind wandering (e.g., “*It feels like I don’t have control over when my mind wanders*;” “*I find my thoughts wandering spontaneously*.”).

##### NEO Conscientiousness scale

We used nine NEO-FFI-3 items (McCrae & Costa Jr., 2010), with three items each representing Dutifulness, Achievement, and Self-Discipline facets. Items asked about dependability, goal striving, and being productive and efficient.

##### NEO Openness scale

We included all 12 NEO-FFI-3 items, with two items reflecting the Fantasy facet, one reflecting Actions, three reflecting Aesthetics, two reflecting Feelings, three reflecting Ideas, and one reflecting Values; it also included the remaining six Fantasy items from the NEO-PI-R (McCrae & Costa Jr., 2010). Items asked about imaginativeness and enjoying daydreams, learning new activities, aesthetic chills and pattern seeking, experiencing and noticing emotions, intellectual curiosity, and exposure to controversial viewpoints.

##### Spontaneous Activity Questionnaire scale (SAQ)

The eight SAQ items from Carriere et al. (2013) asked about fidgeting (e.g., “*I often fidget when I am planning ahead for something*”). Because the original subscale used frequencies as response options, we revised all items to fit a disagree–agree scale (e.g., we revised “*I fidget*” to “*I fidget a lot*”); these revisions yielded five regularly scored items (higher ratings = more fidgeting) and three reverse-scored items.

##### White Bear Suppression Inventory

The 15 items from Wegner and Zanakos (1994) asked about uncontrollable thoughts and avoiding unwanted thoughts (e.g., “*I have thoughts that I cannot stop*;” “*I always try to put problems out of mind*”).

##### Attentive Responding Scale (ARS) Infrequency subscale

Instructions for the questionnaire battery forewarned the inclusion of attention-check items, to minimize backfire effects from odd questions:

In this task, you will answer various questions about your everyday thoughts and experiences. For each question, please rate how strongly you disagree or agree with each statement. A few questions will be odd or silly, as a way to be sure you’re paying attention. Please just answer each question carefully and honestly.

The questionnaire included the six infrequency items (three reverse scored) from the 18-item ARS scale (Maniaci & Rogge, 2014), appearing here for all subjects as questions 31, 41, 71, 81, 131, and 141. These items were created to yield the same answers for all subjects (e.g., “*I don’t like getting speeding tickets*;” “*I enjoy the music of Marlene Sandersfield*;” “*I’d rather be hated than loved*”). In addition, we included two questions that directed subjects to select a response, inspired by the Directed Question Scale (Maniaci & Rogge, 2014): “*I will show I am paying attention by selecting choice Strongly Agree*;” “*To show I’m reading this item carefully, I will select choice Strongly Disagree*.” For all subjects, the directed items appeared as questions 101 and 121. From the six infrequency and two directed items we created an 8-item Infrequency scale.

For each of the original infrequency items, we scored it as a 0 if the subject selected a response on the appropriate side of neutral; we scored it as one point if the subject selected response “3” (Neither agree or disagree) and as two points if the subject selected either response on the inappropriate side of neutral. For each directed question item, we scored it as a 0 if the subject selected the correct response (1 or 5), but as a 1–4 for any erroneous response, reflecting the number of choices away from the correct response. For any skipped item in the Infrequency scale, the subject earned two points. Extrapolating from the cut-offs from Maniaci and Rogge (2014), who used more infrequency items and a slightly different scoring scheme, we dropped all questionnaire data for subjects scoring > 4.

##### ARS Inconsistency subscale

The questionnaire included the six inconsistency pairs from the 18-item ARS, with the paired-items appearing for all subjects as questions 11 and 151, 21 and 161, 51 and 171, 61 and 181, 91 and 191, and 111 and 201. Both members from each pair should yield consistent responses for a given subject, whether or not they endorse the items (e.g., “*I enjoy relaxing in my free time*” and “*In my time off I like to relax*”). We scored each item pair by taking the absolute numerical difference between the two responses; for example, identical choices = 0 and maximally discrepant choices (e.g., Strongly agree to Strongly disagree) = 4. Any skipped item earned two points for that pair. Extrapolating from the endorsed cut-offs (based on more inconsistency items), we dropped all questionnaire data for subjects scoring > 5.

#### Demographic Questionnaire

Subjects reported their sex/gender (via free response), age (via free response), ethnicity ( “*Hispanic or Latino*” or “*not Hispanic or Latino*”), race (“*Asian*,” “*Black: African or Caribbean descent*,” “*Native American or Alaskan Native*,” “*Native Hawaiian or Pacific Islander*,” “*White: European or Middle Eastern descent*,” or “*Multiracial*”) and college major (via free response; we did not analyze these data). A final question asked whether subjects had previously participated in a thought-probe study.

#### Thought probes

Subjects saw one of four probe types in both SART and flanker tasks (i.e., probe type repeated across tasks). All probes asked subjects to characterize their immediately preceding thoughts—in the instant before the probe—via forced-choice response. Our three primary probe types differed in the dimensions of thought assessed: Content probes asked subjects about the topics of their mind-wandering (“*What*”), intentionality probes asked about the reasons for their mind-wandering (“*Why*”), and depth probes asked about the extent or extremity of their mind-wandering (“*How Much*”). A secondary, fourth probe type also assessed thought content, but did not include a content category for thoughts about their task performance (“task-related interference;” TRI).

A second screen asked subjects to rate their confidence in their preceding thought report on a vertically oriented 1–5 scale, labeled on-screen, from top to bottom, as: *1. Not at all confident; 2. Somewhat confident; 3. Confident; 4. Very confident; 5. Extremely confident*. Subjects pressed the keyboard key corresponding to their choice.

Subjects responded to each probe by pressing the key corresponding to one of the numbered options. RTs were recorded but we imposed no time limits. After explaining how probes worked and would appear, the experimenter read these instructions aloud (subjects saw “*category*” in the bracketed text below for content and intentionality probes, and “*response*” in the bracketed text for depth probes):

Remember, when you see a screen like this, please respond based on what you were thinking *just before* the screen appeared. Do not try to reconstruct what you were thinking during the preceding words on the screen, and please select the [category/response] that best describes your thoughts as accurately as you can. Remember that it is quite normal to have any of these kinds of thoughts during an ongoing task.

##### Content probes

Our primary content (“*What*”) probes, with TRI included, provided eight response options, numbered in the following order in a vertically oriented list. The italicized text appeared at each probe, and the experimenter explained the choices via instructions at the beginning of the task: (1) *The task*, for thoughts about task stimuli, required responses, or task goals; (2) *Task experience/performance*, for thoughts about one’s performance or task difficulty (TRI); (3) *Everyday things*, for thoughts about routine events in the recent or distant past or future; (4) *Current state of being*, for thoughts about physical or emotional states; (5) *Personal worries*, for thoughts about life concerns; (6) *Daydreams*, for fantasies or thoughts disconnected from reality; (7) *External environment*, for thoughts about objects or task-unrelated events in the room; (8) *Other*, for any thoughts not captured by the other choices.

Our secondary content probes, with TRI excluded, presented probes exactly like those above, but without response option 2. Otherwise, each of the response options above was numbered from 1 to 7. In both content-probe conditions, the experimenter read these instructions before explaining the thought probes:

It is perfectly normal to think about things that are not related to the task. We will give you several categories of things that people might think about during tasks like these. Please try your best to honestly assess your thoughts and choose a category that best describes your thoughts at the time when we ask.

##### Intentionality probes

Our intentionality (“*Why*”) probes asked whether subjects had been mentally on- or off-task and, if off-task, whether they’d been so intentionally or unintentionally. The experimenter explained TUTs and intentionality by reading these on-screen instructions:

It is perfectly normal to think about things that are not related to the task. For instance, you may think about off-task things such as something you did recently or will be doing later, your current emotional or physical state, personal worries, daydreams, or your external environment. Please try your best to honestly assess your thoughts and choose a response that best describes your thoughts at the time when we ask.

There are generally two paths to off-task thinking. INTENTIONAL: Sometimes when you’re working on a task, you deliberately or intentionally think about things unrelated to the task. This is what we refer to as intentional off-task thinking. That is, when you deliberately think about something other than the task. UNINTENTIONAL: Other times when you’re working on a task, you unintentionally think about things unrelated to the task. This is what we refer to as unintentional off-task thinking. That is, when you spontaneously think about something other than the task.

Each probe presented three numbered response options on-screen, appearing in a vertically oriented list: (1) The task; (2) Off-task: Intentional (on purpose); (3) Off-task: Unintentional (spontaneous).

##### Depth probes

Our depth (“*How Much*”) probes asked subjects to rate the extent to which their immediately preceding thoughts were on- or off-task, using a 1–5 scale. The experimenter explained TUTs by reading these on-screen instructions:

It is perfectly normal to think about things that are not related to the task. For instance, you may think about off-task things such as something you did recently or will be doing later, your current emotional or physical state, personal worries, daydreams, or your external environment. Please try your best to honestly assess your thoughts and choose a response that best describes your thoughts at the time when we ask.

Each probe presented five numbered response options on-screen, appearing in a vertically oriented list: (1) Completely on-task; (2) Mostly on-task; (3) Both on the task and off-task; (4) Mostly off-task; (5) Completely off-task (e.g., Franklin et al., 2011; Mrazek et al., 2012).

### General procedures

Subjects completed a 100–120 min session in groups of 1–4, each at their own workstation (except during two semesters at WCU, where subjects completed only the cognitive tasks in a 60-min session). An experimenter read aloud on-screen instructions and remained to answer questions and monitor behavior. “Wait” screens appeared at the end of each task to prevent subjects from advancing until all subjects finished.

## Results

This study presented a challenge for defining an analytic approach. It was exploratory insofar as we didn’t entertain theoretically derived hypotheses about which probe types might elicit discrepant effects. The study asked a specific question, however—*whether commonly used probe types, which assess distinct dimensions of subjective experience, yield different results*—and a set of outcomes we took to be most important (although not preregistered, we describe most outcomes and analyses in a rudimentary analysis-plan document finalized after data collection but before we analyzed the full dataset, posted along with our data at https://osf.io/vs2u5/.

We took a frequentist approach to analyses—while considering point estimates, confidence intervals, and effect sizes—to provisionally answer our validity questions about probe-types and to prioritize findings as worthy of future replications. In keeping with the exploratory aspect of the study, we erred toward false positives by not applying a correction for multiple comparisons. (Moreover, our analyses addressed many related questions that were not clearly separable to guide familywise error correction.) However, because we conducted many analyses, we exerted some modest control over Type I error by adopting an alpha level of .005 throughout (and we report, where applicable, 99.5% confidence intervals and exact *p* values > .001).

We supplemented some of our frequentist analyses with Bayes factors (BFs). BFs allowed us to compare predictive performance of competing models (Kass & Raftery, 1995), providing a continuous measure of evidence. Our null model reflected a Cauchy distribution (50% of the distribution was between d = −.707 and d = .707). We interpreted BFs < 0.33 as providing modest evidence for the null and BFs > 3.0 as providing modest evidence for the alternative hypothesis, and BFs < .10 as providing strong evidence for the null and BFs > 10 providing strong evidence for the alternative hypothesis.

We originally intended to analyze all four thought-probe conditions together but modified the plan upon preparing a presentation on these data (Kane et al., 2018), which suggested a question-focused organization. Thus, we first report analyses for the three probe conditions addressing our primary questions about valid TUT probing (content probes with TRI included, intentionality probes, and depth probes), and then report analyses for our two content probe conditions to address our secondary questions about TRI.

### Data analysis exclusions

UNCG and WCU students were eligible to enroll if English was their native language, they were 18–35 years old, they were enrolled in introductory psychology, and they had not participated in a thought-probe study. We followed the conservative post-enrollment data exclusion criteria used by Kane et al. (2016) wherever possible. The Appendix describes the criteria used to exclude data based on experimenter reports, missing tasks, outlying task scores, and questionnaire responses, and it specifies the number of subjects whose data were dropped at each stage.

### Final sample demographics

Our final sample of 1067 subjects had a *M* age of 19.0 (SD = 1.9; *n* reporting = 1066). Regarding gender, 63.7% self-identified as female, 36.0% as male, and 0.1% identified in some way as non-binary (*n* reporting = 1065). The racial composition of the sample (*n* reporting = 1041) was Asian = 3.4%, Black (African or Caribbean descent) = 31.4%, Native American or Alaskan Native = 1.1%, Native Hawaiian or Pacific Islander = 0.4%, White (European or Middle Eastern descent) = 55.8%, and multiracial = 6.8%; self-reported ethnicity, asked separately, was 7.2% Latino/a or Hispanic (*n* reporting = 1065).

### Content versus intentionality versus depth probe reports

**Fig. 1:**
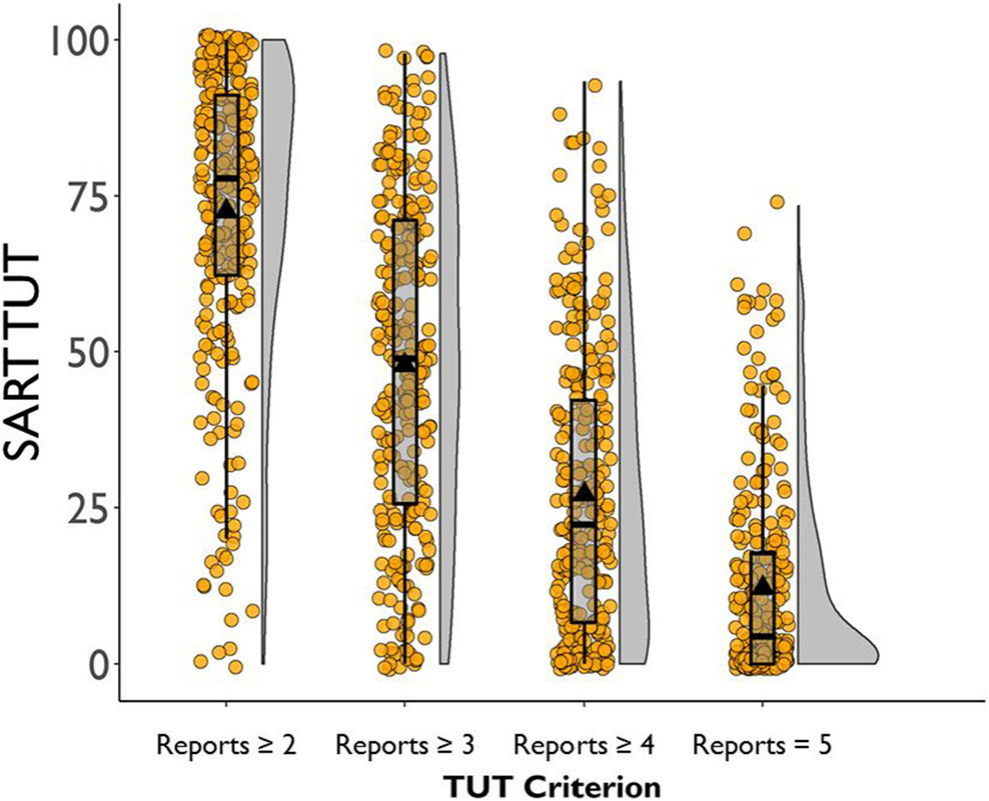
Percent task-unrelated thought (TUT) reports in the Sustained Attention to Response Task (SART) from the depth probe condition, as a function of defining TUTs as thought ratings of ≥ 2, ≥ 3, ≥ 4, or 5. Box plots present the 25^th^, 50^th^, and 75% percentiles; whiskers extend to the smallest and largest values within 1.5 times the inter-quartile range. Means are presented as triangles; circles represent individual subjects’ TUT rates

To allow TUT-rate comparisons across probe types, we translated the 1–5 ratings from the depth condition into a categorical TUT definition that was comparable to the other conditions (subsequent analyses will treat depth ratings as continuous). We established a point along the scale above which a thought should be considered a TUT. The cut-point was the value producing *M* TUT rates from the first task (SART) closest to those from the content and intentionality conditions—these were both close to 50%. Figure 1 shows that defining a TUT as any rating of ≥ 3 (*both on the task and off-task*) yielded approximately a 50% TUT rate average. Subjects in the depth condition thus seemed to treat an evenly split focus of attention (or, perhaps, any probe response choices that had “*off-task*” in the label) in the same way that subjects treated TUT experiences in the content and intentionality probe conditions. Of note, a daily-life study that probed subjects with either 1–5 depth probes or categorical on/off-task probes similarly found that a cut-point of ≥ 3 on depth probes produced matching *M* TUT rates to the categorical condition (37 vs. 40%; Seli, Beaty, et al., 2018).

#### Comparing means and consistency across probe types and tasks

Our first analyses tested whether TUT rates or confidence in TUT reports differed by probe type. Our analyses also tested for any differential consistency of TUT reports and TUT confidence ratings across SART and flanker tasks.

##### Mean TUT rates

**Fig. 2:**
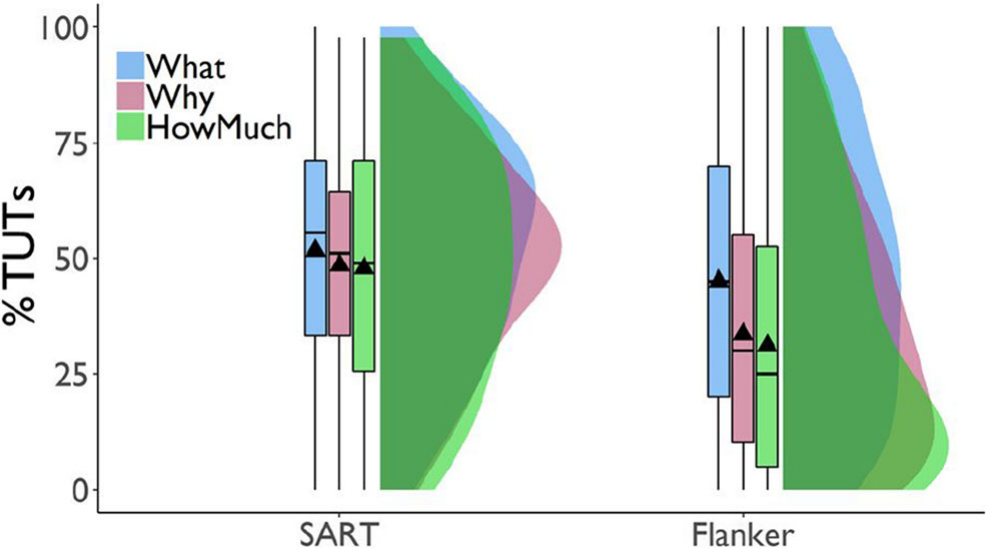
Percentage of task-unrelated thoughts (TUTs) reported in the Sustained Attention to Response Task (SART) and the flanker task, for subjects in the content (“What”), intentionality (“Why”), and depth (“HowMuch”) probe conditions. Box plots present the 25^th^, 50^th^, and 75% percentiles; whiskers extend to the smallest and largest values within 1.5 times the inter-quartile range. Means are presented as triangles

Our most basic questions were whether different probes yielded different TUT rates and whether these showed differential cross-task stability. The data in Fig. 2 suggest that TUT rates were similar across probe types in the SART. TUT rates diverged, however, in the subsequent flanker task, dropping less from the SART to flanker in the content (“What”; *n* = 266) condition than in the intentionality (“Why”; *n* = 263) and depth (“How Much”; *n* = 269) conditions.

We tested these impressions with a linear mixed model (LMM) approach using the lme4 package (Bates et al., 2015). Starting broad then narrowing, we first examined an ANOVA table, then parameter estimates for each level of the factor, and finally all pairwise critical comparisons (made with the Least Square Means package [Lenth, 2016]). We computed *p* values for parameter estimates (i.e., at the second stage of this inferential approach) using the Satterthwaite approximation contained in the lmerTest package (Kuznetsova et al., 2017), which produces *p* values in line with actual false positive rates (Luke, 2017). The model predicted TUT rate with task and probe-type condition as fixed-effect predictors and subjects as the random effect. The ANOVA results indicated that TUT rates were higher in the SART than the flanker task, *F*(1, 795) = 242.71, *p* < .001, TUT rates differed across probe types, *F*(2, 795) = 10.69, *p* < .001, and probe-type interacted with task, *F*(2, 795) = 15.54, *p* < .001.

At the parameter level, we explored the probe-type × task interaction with TUT rate from content probes in the flanker task set as the reference condition. The difference in TUT rates between content and intentionality probe conditions in the flanker task was significantly larger than that in the SART, b = .09, SE = .02, *t*(795) = 4.29, *p* < .001, as was the difference between the content and depth conditions, b = .10, SE = .02, *t*(795) = 5.23, *p* < .001 (although the cross-task changes in TUT rate were significant for all probe-type conditions; for all paired contrasts, *t*s > 4.51, *p*s < .001).

Moreover, in the SART, TUT rates did not differ significantly across probe types (*M*s = .52, .49, .48 for content, intentionality, and depth probes, respectively); for all paired contrasts from the linear mixed effects model, *t*s < 1.65, *p*s > .09. To aid interpretation of these null effects, we calculated BFs from *t* tests for these comparisons using the BayesFactor package (Morey & Rouder, 2018); these analyses yielded BF_10_ = .10, .21, and .30, each indicating data more in favor of the null model than the alternative model.

In the flanker task, however, TUT rates were higher in the content (*M* = .45) than intentionality (*M* = .34) condition, b = 0.12, SE = 0.02, *t*(1142.1) = 5.06, *p* < .001, and depth (*M* = .31) condition, b = .14, SE = .02, *t*(1142.1) = 6.17, *p* < .001; the latter two did not differ, b = 0.02, SE = 0.02, *t*(1142.1) = 1.08, *p* = .283. BFs for the contrasts of content versus intentionality probes (BF_10_ = 3173.37), and content versus depth probes (BF_10_ = 197,320.30) yielded strong evidence favoring the alternative over the null model; for intentionality versus depth probes, however, BF_10_ = .16, providing more support for the null than the alternative model. So, although probe types did not measurably affect TUT rates in the SART, they did in the flanker task (with TUT rates remaining more stable for the content condition across tasks than for the other conditions), our first indication that not all probed assessments of mind-wandering are created equal.

##### TUT rate correlations

We next assessed whether individual differences showed similar cross-task reliability across probe types by correlating SART TUTs with flanker TUTs. All correlations were substantial and significant, with nearly identical effect sizes and 99.5% confidence intervals. For content probes, *r*(264) = .61 [.49, .71], for intentionality probes, *r*(261) = .64 [.53, .73], and for depth probes, *r*(267) = .65 [.54, .74]. We find no evidence that probe type influenced variation in, or cross-task stability of, TUT rates.

##### Mean TUT confidence ratings

**Fig. 3:**
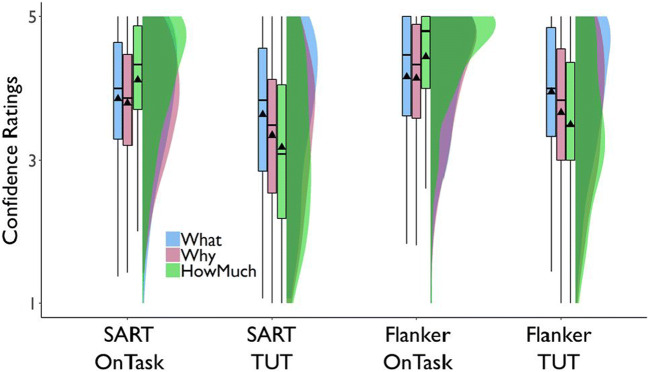
Mean confidence ratings for task-unrelated thought (TUT) and on-task (OnTask) reports in the SART and the flanker task, for subjects in the content (“What”), intentionality (“Why”), and depth (“HowMuch”) probe conditions. Box plots present the 25^th^, 50^th^, and 75% percentiles; whiskers extend to the smallest and largest values within 1.5 times the inter-quartile range. Means are presented as triangles

We next assessed whether confidence in TUT reports varied with probe type, and whether confidence differed in cross-task stability among probe types. We used data for all subjects with at least one TUT report. Figure 3 (which also presents confidence in on-task reports, discussed later) suggests that *M* confidence in TUT reports increased from the SART to flanker task, and subjects more confidently reported the content of TUTs than intentionality or depth. The ANOVA table results indicated that TUT confidence ratings were higher in the flanker than the SART, *F*(1, 711.7) = 99.45, *p* < .001, and confidence differed across probe types, *F*(2, 779.0) = 16.17, *p* < .001; they did not, however, produce a significant probe-type × task interaction, *F*(1, 711.5) = 0.42, *p* = .658.

Paired contrasts from the LMM indicated that, in the SART, TUT confidence was significantly greater for content than intentionality probes, b = 0.29, SE = 0.09, *t*(1010.2) = 3.11, *p* = .002, and depth probes, b = 0.45, SE = 0.09, *t*(1008.9) = 4.90, *p* < .001; the latter two did not differ significantly, b = 0.16, SE = 0.09, *t*(1010.3) = 1.77, *p* = .076. Again, to strengthen interpretation of these effects, we calculated BFs from *t* tests for these comparisons. BFs for the contrasts of content versus intentionality probes (BF_10_ = 18.93), and content versus depth probes (BF_10_ = 25,566.20) indicated strong evidence favoring the alternative over the null model; for intentionality versus depth probes, however, BF_10_ = .54, providing weak support for the null over the alternative model.

Similarly, in the flanker task, TUT confidence was significantly greater for content than intentionality probes, b = 0.30, SE = 0.09, *t*(1064.2) = 3.24, *p* = .001, and depth probes, b = 0.52, SE = 0.09, *t*(1089.1) = 5.45, *p* < .001; the latter two did not differ significantly from each other, b = 0.21, SE = 0.10, *t*(1108.3) = 2.21, *p* = .028. BFs for the contrasts of content versus intentionality probes (BF_10_ = 32.01), and versus depth probes (BF_10_ = 27,204.33) indicated strong evidence favoring the alternative over the null model; for intentionality versus depth probes, however, BF_10_ = .40, providing weak support for the null over the alternative model. Like TUT rates, then, confidence ratings suggest some differences among probe types, here with consistently higher confidence in TUT reports for the content condition than the other conditions.

We next considered confidence in TUT versus on-task thought reports. Figure 3 suggests that the content condition yielded more similar confidence ratings between on-task and TUT reports than did intentionality or depth conditions. We tested these impressions by creating a difference score for each subject in each task, subtracting TUT from on-task confidence, and submitting these difference scores to a LMM with the flanker content condition as the reference level. Compared to the content condition, the confidence difference scores in the flanker task were significantly larger in the intentionality condition, b = 0.29, SE = 0.09, *t*(1203) = 3.24, *p* = .001, and in the depth condition, b = 0.78, SE = .09, *t*(1247) = 8.45, *p* < .001; somewhat similarly, in the SART, the content condition yielded a (non-significantly) smaller difference score than did the intentionality condition, b = 0.22, SE = .09, *t*(1129) = 2.60, *p* = .009, and a significantly smaller difference score than did the depth condition, b = 0.71, SE = .09, *t*(1140) = 8.14, *p* < .001. The depth condition produced still larger confidence difference scores than did the intentionality condition in both the flanker, b = 0.49, SE = 0.09, *t*(1238) = 5.35, *p* < .001, and SART, b = 0.48, SE = 0.08, *t*(1130) = 5.59, *p* < .001. Overall, then, subjects were more confident about their content-based TUT reports than their intentionality- or depth-based TUT reports, and they were more similarly confident in TUT and on-task reports in the content condition than the other conditions; the largest discrepancies between on- and off-task confidence reports were produced in the depth condition.

##### Confidence ratings correlations

We next tested whether confidence ratings showed similar cross-task consistency for the three probe types by correlating SART with flanker TUT confidence. Again, all correlations were similar. For content probes, *r*(247) = .74 [.65, .81], for intentionality probes, *r*(227) = .72 [.62, .80], and for depth probes, *r*(215) = .67 [.55, .76]. We found no support for probe types measurably affecting individual differences in, or reliability of, confidence reports for TUT experiences.

##### Summary of cross-task comparisons

Within the limits of our methods and analytic approaches, we found no statistical evidence for probe-type differences in SART TUT rates (note the depth probe TUT rate in the SART was set to match those from the content and intentionality rates), in TUT-rate correlations between SART and flanker tasks, or in TUT confidence correlations between tasks. We did find, however, statistical evidence for the following differences: 1) TUT rates for intentionality and depth probes dropped more from the SART to flanker task than they did for content probes; in the flanker task, content probes yielded more than 10% higher TUT rates than the intentionality or depth probes. 2) In both tasks, subjects reported TUTs with more confidence in the content condition (reporting on the “*what*” of their experience) than in the intentionality condition (reporting on the “*why*”) and in the depth condition (reporting on “*how much*”), with depth confidence ratings in TUT about a half point lower (on a five-point scale) than in the content condition; confidence reports in the content condition were also more similar between on- and off-task reports than were those in the intentionality or depth conditions.

#### Comparing within-person SART performance correlates of TUTs across probe types

To test whether in-the-moment performance differentially correlated with in-the-moment TUT reports across probe conditions, we focused on the SART because it presented enough trials to allow within-person analyses and presented only one trial type for RTs. We first examined RT variability (“RTsd”)— the standard deviation across the four “go” trials preceding each no-go trial and thought report—to test whether the predicted increase in RTsd before TUTs versus on-task reports differed across probe types. We next examined no-go trial accuracy preceding each thought report to test whether the predicted increase in no-go errors preceding TUTs versus on-task reports differed across probe types.

##### RT variability

**Fig. 4:**
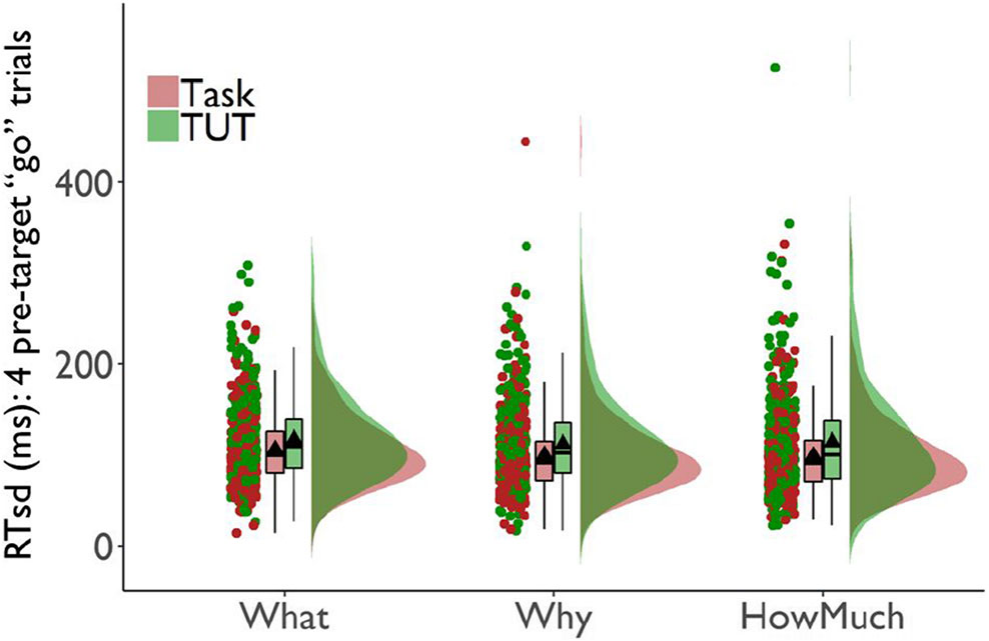
Standard deviations in reaction times (RTsd) for the four go-trials preceding task-unrelated thought (TUT) reports versus on-task (Task) reports in the SART, for subjects in the content (“What”), intentionality (“Why”), and depth (“HowMuch”) probe conditions. Box plots present the 25^th^, 50^th^, and 75% percentiles; whiskers extend to the smallest and largest values within 1.5 times the inter-quartile range. Means are presented as triangles. Each dot represents an individual subject’s RTsd preceding TUT or on-task reports

Figure 4 suggests that our study replicated prior findings (e.g., Bastian & Sakur, 2013; Seli, Cheyne, & Smilek, 2013): Subjects’ go-trial RTs were slightly more variable preceding TUT reports than on-task reports. Notably, this TUT-related increase in RTsd was similar across probe conditions. For the content-probe condition, *M*s = 106 ms (SD = 39) vs. 118 ms (SD = 48) preceding on-task versus TUT reports, respectively; for the intentionality condition, *M*s = 100 ms (SD = 45) vs. 113 ms (SD = 49), and for the depth condition, *M*s = 99 ms (SD = 42) vs. 115 ms (SD = 62).

A LMM with on-task reports in the content-probe condition as the reference level indicated that RTsd was greater preceding TUTs than on-task reports there, b = 8 ms, SE = 2 ms, *t*(20686.3) = 3.66, *p* < .001. This small RTsd difference did not differ significantly for the content versus intentionality condition, b = 3 ms, SE = 3 ms, *t*(20794.4) = 0.91, *p* = .361, or depth condition, b = 7 ms, SE = 3 ms, *t*(20483.2) = 1.98, *p* = .047. The RTsd difference preceding TUT vs. on-task reports was significant in each probe condition; all *t*s > 3.65, *p*s < .001. (Fig. 4 indicates some outliers; conservatively, we did not delete them because: (a) we already dropped data from 13 subjects with outlying SART RTsd; (b) Fig. 4 shows that the pattern held across intentionality and depth conditions despite their having outliers in opposite cells (on-task vs. TUT) and; (c) Fig. 4 shows increased RTsd preceding TUT reports throughout the tails of the TUT distributions.)

##### No-go trial accuracy

**Fig. 5:**
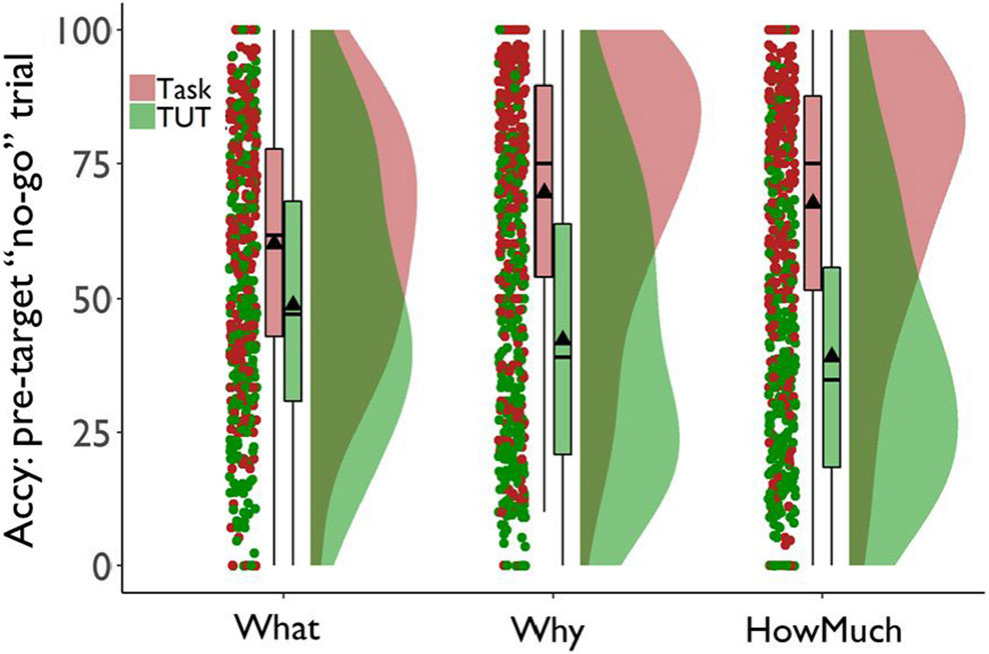
Accuracy (“Accy”) rates for no-go trials preceding task-unrelated thought (TUT) reports versus on-task (Task) reports in the SART, for subjects in the content (“What”), intentionality (“Why”), and depth (“HowMuch”) probe conditions. Box plots present the 25^th^, 50^th^, and 75% percentiles; whiskers extend to the smallest and largest values within 1.5 times the inter-quartile range. Means are presented as triangles. Each dot represents an individual subject’s *M* no-go accuracy rate preceding TUT or on-task reports

The data presented in Fig. 5 show clearly that no-go trial accuracy was poorer preceding TUT than on-task reports (replicating, e.g., McVay & Kane, 2009, 2012a). In contrast to what we found in RTsd, however, the difference between on-task and TUT-reported trials varied dramatically across probe conditions; the different distributions clearly pass the “interocular trauma test” (hitting one between the eyes). For the content-probe condition, *M*s = 59.9% (SD = 23.3) vs. 48.8% (SD = 24.8) preceding on-task versus TUT reports, respectively; this large effect was nonetheless dwarfed by that in the intentionality condition, *M*s = 69.6% (SD = 24.8) and 42.4% (SD = 25.8), and the depth condition, *M*s = 67.7% (SD = 26.5) and 39.2% (SD = 25.2). We analyzed accuracy using a generalized linear mixed model (GLMM) in the lme4 package (Bates et al., 2015) to account for the binomial distribution of trial-level accuracy (Dixon, 2008). A GLMM with on-task reports in the content-probe condition set as the reference level indicated that no-go trial accuracy was significantly lower preceding TUTs than on-task reports there, b = −.56, SE = .05, Z = −12.45, *p* < .001. This large accuracy difference between TUT and on-task reports was nonetheless significantly smaller than in the intentionality-probe condition, b = −.93, SE = .07, Z = −13.88, *p* < .001, and depth-probe condition, b = −.90, SE = .07, Z = −13.40, *p* < .001.

##### Summary of within-person performance correlates

In all probe-type conditions, RT variation increased (modestly), and accuracy decreased (markedly), preceding TUT reports compared to on-task reports. Notably, however, whereas RTsd showed similar effects across probe types, accuracy did not. Intentionality and depth reports distinguished commission errors from stopping more sharply than did those to content probes. That is, immediately after erroneously responding to a no-go stimulus, subjects in the intentionality and depth conditions were more likely than those in the content condition to report TUTs, and after correctly stopping they were more likely to report having been on-task.

#### Comparing correlations of TUT rates to retrospective mind-wandering reports

The following analyses tested whether probed TUT reports differentially correlated with post-task questionnaire (DSSQ) measures of task-unrelated mind wandering across probe-type conditions. As expected, all TUT–DSSQ correlations were substantial and significant (but not enough to suggest redundancy), with similar effect sizes and overlapping 99.5% confidence intervals. TUT–DSSQ correlations from the SART were numerically somewhat variable: for content probes, *r*(225) = .32 [.14, .48], for intentionality probes, *r*(219) = .43 [.26, .57], and for depth probes, *r*(222) = .36 [.19, .51]; however, even the largest difference among these (content vs. intentionality probes), was not statistically significant, z = 1.31, *p* = .19. Correlations from the flanker task were all nearly identical: for content probes *r*(225) = .40 [.23, .54], for intentionality probes, *r*(219) = .36 [.18, .51], and for depth probes, *r*(222) = .36 [.19, .51]. Subjects who retrospectively reported on the DSSQ that they had mind-wandered more during a task also tended to report more probed TUTs during the task, regardless of thought-probe type.

#### Comparing correlations of TUT rates to executive-control abilities

One of this study’s primary questions was whether the typical negative correlation between executive-control abilities and TUT rates (e.g., McVay & Kane, 2009, 2012a, 2012b; Robison & Unsworth, 2017, 2018; Unsworth & McMillan, 2013, 2014) varied with probe type. Most prior individual-differences studies have used content-based probes.

##### Establishing an executive-control performance factor

We first derived an executive control latent variable, for all subjects, from antisaccade-letter accuracy, antisaccade-arrow accuracy, SART d′, and SART RTsd. We did so by conducting a multigroup confirmatory factor analysis (CFA), with groups corresponding to our four probe conditions (*N*s = 263–269). We assessed measurement invariance with the semTools package to assess measurement invariance (Jorgensen et al., 2018) and lavaan for the multigroup CFA (Rosseel, 2012). The single-factor CFA model was loaded by all four measures and included a residual correlation between the two SART measures (following Kane et al., 2016). The baseline, configural model adequately fit the data for all four groups, indicated by a non-significant Chi-square test, χ^2^(4) = 2.77, *p* = .596; in all four groups, all measures loaded significantly on the executive-control factor. Table 1 presents chi-square tests (along with AIC and BIC fit statistics) indicating measurement invariance across groups: Constraining factor loadings to be equal across groups (“weak invariance”) did not significantly hurt model fit versus the configural model, constraining intercepts to be equal across groups (“strong invariance” or “scalar invariance”) did not hurt model fit versus the weak invariance model, and constraining latent-variable means to be equal across groups did not hurt model fit versus the strong invariance model.

**Table 1:** Test for measurement invariance with multigroup confirmatory factor analysis

Models	Df	AIC	BIC	χ^2^	χ^2^ diff	Df diff	*p*
Configural Model	4	11032	11291	2.73			
Constrain Loadings	13	11023	11236	11.39	8.66	9	0.469
Constrain Intercepts	22	11005	11174	11.41	0.02	9	1.000
Constrain Means	25	10999	11153	11.41	0.00	3	1.000

We therefore applied the single-factor executive-control model to the full sample and saved subjects’ factor scores as an indicator of executive-control performance. The model provided an adequate fit to the data: χ^2^(1) = 0.53, *p* = .466, CFI = 1.00, RMSEA = .000, SRMR = .003. Table 2 presents the correlations among the four measures in the full sample and their standardized factor loadings on the executive factor.

**Table 2:** Factor loading and bivariate correlations for the executive control measures in the full sample

Measures	Factor loading	ANTI-LET	ANTI-ARO	SART d′
ANTI-LET	.85			
ANTI-ARO	.72	.61		
SART d′	.43	.37	.30	
SART rtsd	.48	.41	.35	.52

*ANTI-LET* antisaccade letters task, *ANTI-ARO* antisaccade arrows task, *SART* sustained attention to response task, *rtsd* intraindividual standard deviation of response time

##### Correlating executive control with probed TUT rate

Our main question was whether executive control similarly predicted TUT rate across probe types, in both tasks. Correlations are presented in Table 3. For the SART, all correlations were negative; for subjects responding to intentionality probes, the correlation was not significant (*p* = .011; BF_10_ = 3.39). Despite differential significance across probe-type conditions, the TUT-executive correlation from content probes was not significantly larger than that from intentionality probes, z = 2.03, *p* = .042. For the flanker task, all correlations were again negative, but here the correlations were non-significant for both the intentionality-probe group (*p* = .518; BF_10_ = 0.17) and depth-probe group (*p* = .086; BF_10_ = 0.60). The correlation for the content group was numerically smaller than in the SART, but it was still significant and of typical magnitude (*r* = −.22). As we found with the SART, however, the suggestive difference in TUT–executive correlation between the content- and intentionality-probe condition was not statistically significant, z = 2.11, *p* = .035.

**Table 3:** Correlations (with 99.5% confidence intervals) between individual-differences predictor constructs and task-unrelated thought (TUT) rates, in the SART and flanker task, across probe-type conditions

Predictor	Outcome	Content (“What”) Probes	Intentionality (“Why”) Probes	Depth (“HowMuch”) probes
Executive control				
	SART TUTs	*r*(264) = −.32 [−.47, −.16]	*r*(261) = −.16 [−.32, .01]	*r*(267) = −.22 [−.38, −.05]
	Flanker TUTs	*r*(264) = −.22 [−.38, −.05]	*r*(261) = −.04 [−.21, .13]	*r*(267) = −.10 [−.27, .07]
Distractibility and restlessness				
	SART TUTs	*r*(205) = .06 [−.14, .25]	*r*(199) = .25 [.06, .43]	*r*(203) = .08 [−.12, .27]
	Flanker TUTs	*r*(205) = .21 [.02, .39]	*r*(199) = .28 [.09, .45]	*r*(203) = .11 [−.09, .30]
Positive-constructive daydreaming				
	SART TUTs	*r*(205) = .06 [−.14, .25]	*r*(199) = .29 [.10, .46]	*r*(203) = .03 [−.17, .22]
	Flanker TUTs	*r*(205) = .18 [−.01, .36]	*r*(199) = .30 [.11, .47]	*r*(203) = .17 [−.03, .35]

Executive control reflects factor scores from a confirmatory factor analysis of four cognitive performance measures (see text for details); Distractibility and restlessness reflects z-score composites of seven questionnaire measures (see text for details); Positive-constructive daydreaming reflects z-score composites of four questionnaire measures (see text for details)

In summary, we found some non-significant probe-type differences in TUT correlations with executive abilities. Our strict alpha, and BFs reflecting inconsistent evidence for null and alternative hypotheses, discourage strong conclusions about these differences. However, we planned our sample sizes to yield precise estimates of correlation effect size, and only the content-probe condition produced TUT–executive correlations matching typical magnitudes. We recommend future replication efforts on executive-control correlations with TUTs from content versus intentionality probes, particularly across multiple tasks.

#### Comparing correlations of TUT rates to questionnaire measures

In addition to testing whether probe type affected TUT-rate correlations with performance, we also assessed whether they affected TUT-rate correlations with self-report measures of related constructs, such as openness or propensity for cognitive failures (e.g., Carriere et al., 2013; Kane, Gross et al. 2017; McVay & Kane, 2009; Singer & Antrobus, 1970). To do so, we first tried to reduce the number of analyzed constructs.

##### Simplifying the questionnaire battery into fewer constructs

To simplify the 16 questionnaires (with the ADHD questionnaire yielding two outcomes) into fewer variables, we planned to conduct an exploratory factor analysis within each probe-type condition and, assuming similar outcomes, formally testing the simpler structure for factor invariance. Supplemental Table 1 presents correlations among questionnaire measures (along with SART and flanker TUT rates) combined across all four probe-type conditions and then separately for each (*n*s= 201–209 per condition); supplemental Tables 2–5 present exploratory factor analyses for each probe condition and a multi-group CFA to assess factor invariance.

Although many correlations were robust across conditions (e.g., IPI–Daydreaming × Mind-wandering–Deliberate; IPI–Mind-wandering × CFQ–MAL), many others were not. Of 58 correlations with *r* ≥ .30 in the combined dataset, only 32 (55%) correlated with *r* ≥ .30 in all four probe conditions; so, although most correlations were in the same direction across conditions, their magnitudes varied. Factor analyses produced only somewhat similar two-factor solutions across probe conditions: one factor was characterized by low conscientiousness and high inattentiveness, and one by openness and purposeful daydreaming, but also racing and unwanted thinking. Most of the core indicators of each factor spanned conditions, but relative factor loadings varied by condition and more peripheral indicators changed loadings substantially across conditions. Even when we used only a subset of the questionnaire measures that showed the most consistent factor loadings across conditions, we could not demonstrate factor invariance (see Supplemental Table 5).

We therefore changed course and focused our analyses on two subsets of measures that correlated reasonably consistently across conditions and reflected narrower constructs: *distractibility and restlessness*, without the conscientiousness dimension indicated by the two-factor solution described above, and *positive-constructive daydreaming* tendencies, without the racing and unwanted thinking dimensions indicated by the two-factor solution above (see McMillan et al., 2013). We created a z-score composite variable representing distractibility and restlessness from the following measures: ADHD–Hyperactivity, ADHD–Inattentiveness, CFA–MAL, Fidgeting–SAQ, IPI–Boredom Proneness, IPI–Mind Wandering, and Mind Wandering–Spontaneous. We created a z-score composite representing positive-constructive daydreaming from: IPI–Daydreaming, IPI–Problem Solving Daydreams, Mind Wandering–Deliberate, and Openness.

##### Correlating self-reported distractibility and restlessness with probed TUT rate

The correlations between vulnerability to distractibility-restlessness and TUT rate from the SART and flanker tasks, across probe-type conditions, are presented in Table 3. For the SART, the only significant positive correlation was in the intentionality probe condition; the content and depth conditions produced near-zero correlations, *p*s = .377 and .270, BF_10_s = 0.24 and 0.29, respectively. Despite differential significance across conditions, the correlation from intentionality probes was not significantly larger than that from content probes, z = 1.92, *p* = .055. All positive correlations for the flanker task were numerically stronger than for the SART, and here they were significant for both the content- and intentionality-probe groups (for the depth-probe group, *p* = .124, BF_10_ = 0.51). However, the modest difference in correlations between the intentionality and depth conditions was not statistically significant, z = 1.84, *p* = .066.

##### Correlating positive-constructive daydreaming propensity with probed TUT rate

Table 3 presents similar cross-condition, cross-task patterns. For the SART, the only significant correlation was, again, for intentionality probes; for the near-zero correlations for content and depth probes, *p*s = .371 and .640, BF_10_s = 0.24 and 0.18, respectively. Despite differential significance across conditions, the correlations from intentionality and depth probes did not differ, z = 2.61, *p* = .009, given our conservative alpha. The flanker task yielded numerically stronger correlations than did the SART, but again the only significant correlation was for intentionality probes; for the content- and depth-probe groups, *p*s= .010 and .015, BF_10_s = 4.13 and 2.82, respectively). The modest difference in correlation magnitude between intentionality and depth conditions, however, was not significant, z = 1.42, *p* = .156.

In summary, and in parallel to our findings regarding executive control, we found suggestive (but non-significant) probe-type differences in TUT correlations with self-report composites for both distractibility–restlessness and positive-constructive daydreaming. As noted previously, the statistical evidence prevents strong conclusions about any apparent differences, but most BFs for content- and depth-probe correlations indicated substantial evidence for the null hypothesis. Plus, our sample sizes allowed reasonably precise estimates of effect size and only the intentionality-probe TUT rates produced consistently significant correlations (*r*s ≈ .25–.30) with self-report constructs. Our later discussion will consider whether the correlations for the intentionality condition imply validity or some contamination from bias. Regardless, we recommend future replication efforts to focus on these “negative” and “positive” mind-wandering-related constructs, particularly on their correlations with TUT rates from content versus intentionality probes.

### What do TUT depth reports actually measure?

Having established a few differences in TUT reports and their correlates across probe types, we next ask whether TUT depth reports—where subjects rate their extent of on- versus off-task thinking along a scale—provide any more valid information than do dichotomous on-/off-task reports.^3^ Subjects will use a scale if asked, dutifully selecting responses from 1 to 5, but that does not imply that a rating of “2” (labeled *Mostly On-Task*), for example, reflects a conscious state that is different from that reflected by a rating of “3” (labeled *Both On- and Off-Task*). We simply don’t know whether subjects can distinguish being mostly but not completely on-task, from being equally on- and off-task, from being mostly but not completely off-task, or any of these from being completely on- or completely off-task. Even if these assumed intermediate conscious states exist in nature (e.g., Zanesco, 2021), at least sometimes for some people, we should question whether naïve subjects can reliably report on them, and we should question whether graded experience applies equally toward both poles of the continuum. We are concerned that the use of scales to assess mind-wandering has proceeded despite a lack of theoretical or empirical work to characterize the continuum of experience that should inform them (see Tay & Jebb, 2018).

#### Intermediate scale responses as low-confidence reports

Our concerns about the validity of graded TUT reports also stem from the literature on people’s use of middle response options in rating scales to indicate ambivalence, indifference, or incompetence rather than intermediate states (e.g., DuBois & Burns, 1975; Edwards, 1946; Kulas & Stachowski, 2009; Shaw & Wright, 1967), including in self-reports during cognitive tasks. A large metacognition literature has asked subjects to make judgments of learning (JOLs), on a scale of 0–100, reflecting the likelihood they will recall each to-be-learned item on a later test (Metcalfe & Dunlosky, 2008; Nelson & Narens, 1990). These ratings, like TUT reports, should reflect subjects’ evaluations of their conscious states (along with the stimulus context and folk beliefs). In a paired-associate learning study, for example, a subject might provide a JOL of 80% for a word pair that feels easy to remember (e.g., *BAT-ball*) versus JOLs of 50% and 20% for pairs that seem of medium (e.g., *PEN-story*) and high (e.g., *DOG-fork*) difficulty.

How do subjects make JOLs? They may search for evidence for recall and then increment their JOL from 0 upwards as evidence accumulates, with lower JOLs signifying less evidence; here, intermediate ratings indicate intermediate odds of recall, much like a meteorologist uses a “50% chance of rain” to express an intermediate objective probability. Or, subjects may instead use extreme JOLs (e.g., 10%, 90%) to convey high confidence about future performance and intermediate values (e.g., 50%) to convey low confidence; here, intermediate ratings indicate uncertainty, much like a student uses a “50% chance of passing this course” to express reservation about the outcome (for evidence of this “middle response” reporting style in questionnaires, see DuBois & Burns, 1975; Kulas & Stachowski, 2013; Presser & Schuman, 1980). To adjudicate between these possibilities, Dunlosky et al. (2005) had subjects rate their confidence after each JOL. Confidence judgments followed a U-shaped curve, with highest confidence for extremely low and high JOLs, and lowest confidence for intermediate JOLs. Thus, subjects used the middle of the scale not to indicate intermediate odds, but rather to indicate low confidence (see also Serra et al., 2008).

#### Confidence in depth reports

**Fig. 6:**
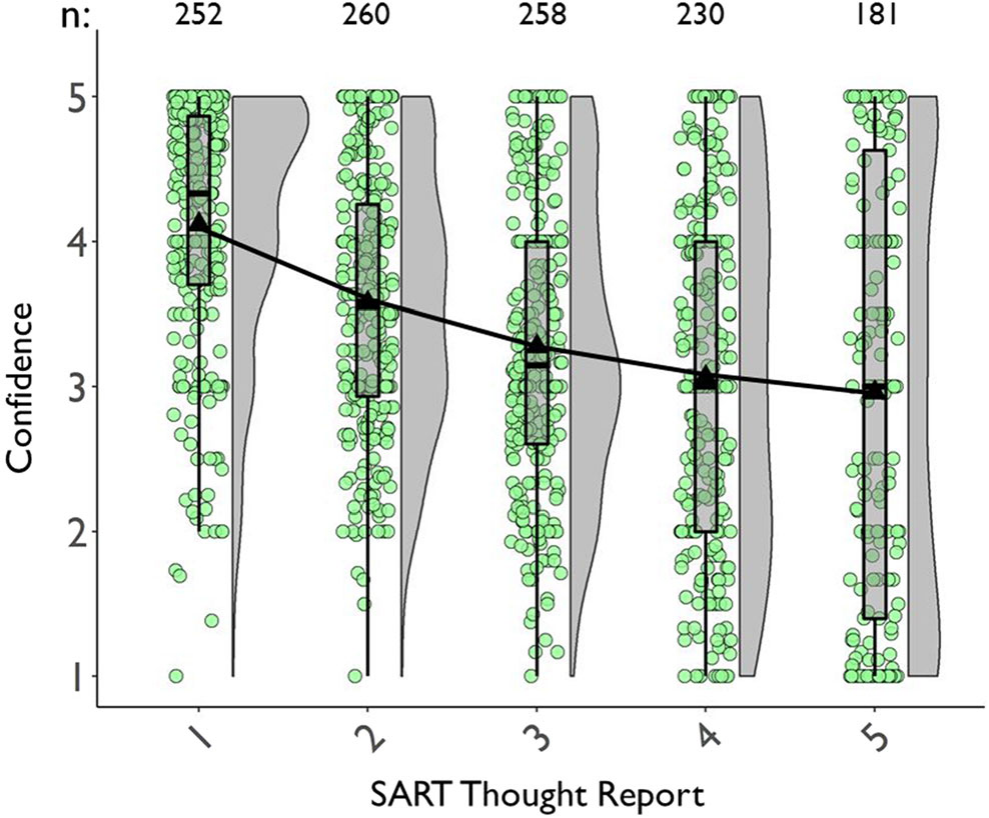
Mean confidence ratings for each depth probe response in the SART; the depth report response scale was labeled: (1) Completely on-task; (2) Mostly on-task; (3) Both on the task and off-task; (4) Mostly off-task; (5) Completely off-task. Box plots present the 25^th^, 50^th^, and 75% percentiles; whiskers extend to the smallest and largest values within 1.5 times the inter-quartile range. Means are presented as triangles. Each dot represents an individual subject’s M confidence rating for that depth rating. Sample sizes varied across depth responses because not every subject used every response scale option

**Fig. 7:**
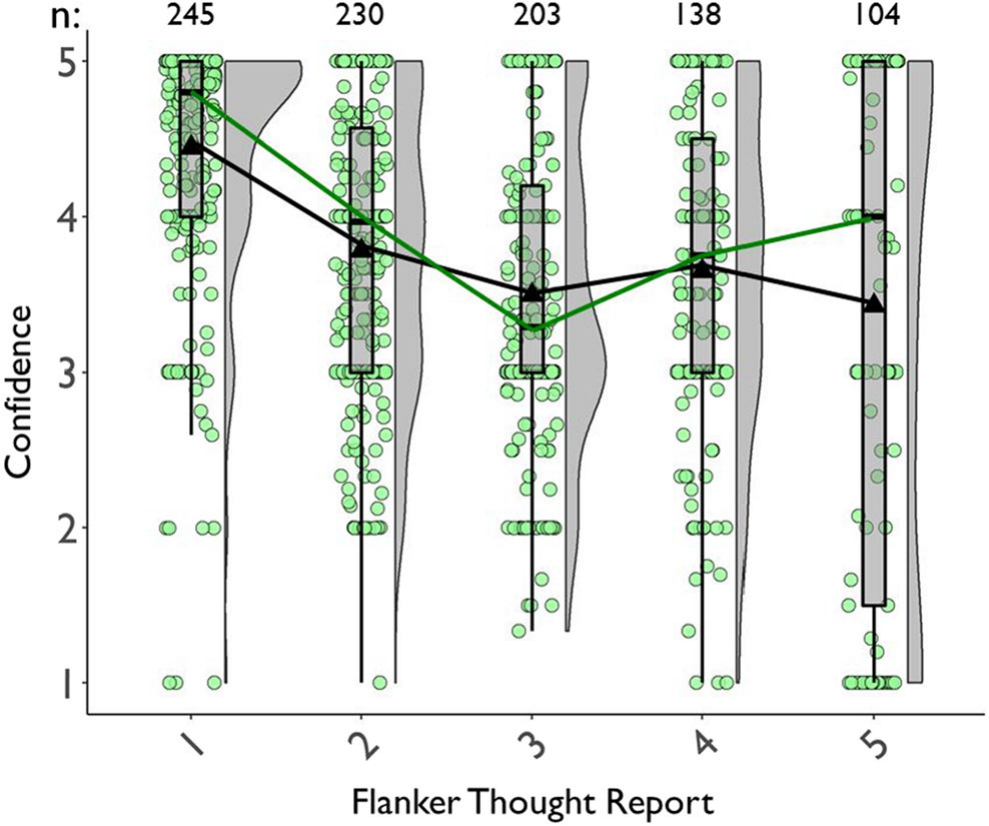
Mean confidence ratings for each depth probe response in the flanker task; the depth report response scale was labeled: (1) Completely on-task; (2) Mostly on-task; (3) Both on the task and off-task; (4) Mostly off-task; (5) Completely off-task. Box plots present the 25^th^, 50^th^, and 75% percentiles; whiskers extend to the smallest and largest values within 1.5 times the inter-quartile range. Means are presented as triangles; black lines connect means and green lines connect medians. Each dot represents an individual subject’s M confidence rating for that depth rating. Sample sizes varied across depth responses because not every subject used every response scale option

Do similar processes drive TUT-depth reports, which are becoming increasingly prevalent (e.g., Allen et al., 2013; Brosowsky et al., 2021; Christoff et al., 2009; Franklin et al., 2011; Konishi et al., 2017; Krimsky et al., 2017; Laflamme et al., 2018; Mills et al., 2018; Mrazek et al., 2012; Ostojic-Aitkens et al., 2019; Seli, Beaty, Cheyne, et al., 2018; Seli et al., 2014; Wammes & Smilek, 2017)? Perhaps subjects use the anchors of the five-point scale to indicate high confidence in their on- versus off-task report and the middle of the scale to indicate low confidence, rather than indicating purer versus more blended experiences of on- versus off-task thought. We examined confidence ratings for each level of thought report on the depth scale, in both the SART (Fig. 6) and the flanker task (Fig. 7), with numerically higher ratings reflecting more confidence.

Confidence in the SART showed an unexpected decrease across increasing ratings—as subjects reported being more off-task they did so with less confidence. But note the bimodal distributions of confidence at depth reports 4 (*Mostly off-task*) and 5 (*Completely off-task*), with most subjects reporting either high or low confidence for more extreme off-task reports. We will return to this pattern shortly. In the flanker task, the *M* confidence ratings show a flatter decrease across increasing off-task ratings and the medians, particularly, show a U-like pattern reminiscent of the JOL data reported by Dunlosky et al. (2005). Depth reports seem to be confounded with confidence. As well, flanker depth ratings of 5 show bimodality, with some subjects showing high confidence and others showing low confidence for extreme off-task reports.

**Fig. 8:**
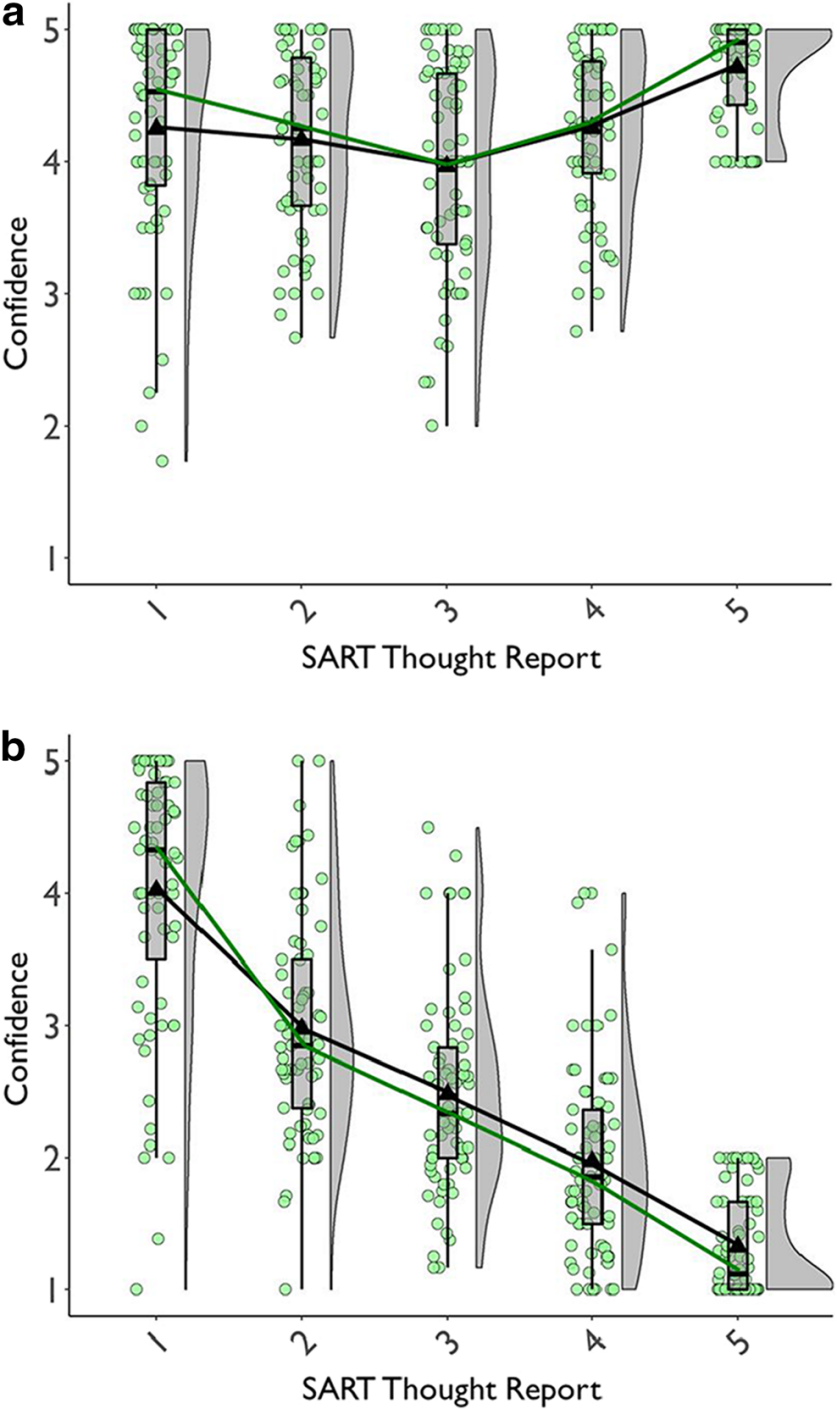
**a** Mean confidence ratings for each depth probe response in the SART for high confidence subjects, who rated their confidence as 4 or 5 (out of 5) for depth ratings of 5 (ns = 61–65 per confidence rating). **b** Mean confidence ratings for each depth probe response in the SART for low confidence subjects, who rated their confidence as 1 or 2 (out of 5) for depth ratings of 5 (ns = 67–70 per confidence rating). For both panels, the depth report response scale was labeled: (1) Completely on-task; (2) Mostly on-task; (3) Both on the task and off-task; (4) Mostly off-task; (5) Completely off-task. Box plots present the 25th, 50th, and 75% percentiles; whiskers extend to the smallest and largest values within 1.5 times the inter-quartile range. Means are presented as triangles; black lines connect means and green lines connect medians. Each dot represents an individual subject’s M confidence rating for that depth rating

To explore the bimodal confidence distributions for TUT ratings of 5 (“*Completely off-task*”), we plot confidence by thought report for two groups in the SART, where we have the most observations: those expressing high confidence (confidence ≥ 4) in off-task depth ratings of 5 (*n* = 65; approximately one-third of the sample) versus those expressing low confidence (confidence ≤ 2) in off-task ratings of 5 (*n* = 70; approximately one-third of the sample). The high-confidence subjects in Fig. 8a show a pattern reminiscent of the flanker-task data and the Dunlosky et al. (2005) data, especially in median ratings, with highest confidence for extreme high (on-task) and low (off-task) depth ratings and lower confidence for scale-midpoint ratings. The most confident third of the sample, then, seemed to subtly confound their reports of conscious states with communications of confidence. In contrast, and curiously, the low-confidence subjects in Fig. 8b show a pattern like the overall SART data, with steadily and starkly decreasing confidence with increasing depth ratings. The least confident third of the sample then, was only highly confident of their “*Completely on-task*” reports, with all subsequent depth ratings eliciting mean and median confidence ratings at the scale midpoint or lower. These subjects may either have relatively poor consciousness-monitoring abilities or, like the high confidence subjects, they may have confounded depth with confidence ratings, here (mis)using increasing values on the depth scale to indicate increased uncertainty about their conscious states.

#### Predictive power of continuous depth reports measures

Neither extreme pattern of confidence ratings—never mind their *mixture*—inspires our confidence in TUT depth reports. The high-confidence pattern indicates a subtle depth-confidence confound and the low-confidence pattern suggests either deficient conscious monitoring or a different variety of depth-confidence confound. Despite our concerns about validity and interpretability, however, we can still ask whether depth ratings provide any useful statistical information beyond that provided by dichotomous on-/off-task reports or scoring. We do this in two ways, one via between-person analyses and another via within-person analyses.

First, in a between-person analysis, we tested whether depth ratings treated continuously show stronger correlations than in our prior analyses with dichotomized depth reports. For each subject we calculated both their *M* depth rating and the variability (SD) in their depth ratings, the latter following up on findings from a small sample that depth rating SDs correlated with some dependent measures from a go/no-go task (Allen et al., 2013). Table 4 presents correlations for TUT depth reports treated continuously (from 1–5) versus treated dichotomously (with TUT = depth reports ≥ 3, as reported from our previous analyses).

**Table 4:** Correlations (with 99.5% confidence intervals) for thought-report ratings from subjects in the depth-probe condition, with thought reports treated either as continuous 1–5 ratings (first for subjects’ mean ratings and second for subjects’ standard deviation of ratings) or as dichotomized on-task versus off-task reports, in the SART and flanker task

Correlation	Depth rating continuous (1–5), rating *M*	Depth rating continuous (1–5), rating SD	Depth rating dichotomized TUT rate (rating ≥ 3 = TUT)
SART × Flanker Rating	*r*(267) = .65 [.54, .74]	*r*(267) = .52 [.38, .63]	*r*(267) = .65 [.54, .74]
SART Rating × SART DSSQ	*r*(222) = .36 [.19, .51]	*r*(222) = .23 [.05, .40]	*r*(222) = .36 [.19, .51]
Flanker Rating × Flanker DSSQ	*r*(222) = .33 [.15, .49]	*r*(222) = .31 [.13, .47]	*r*(222) = .36 [.19, .51]
SART Rating × Executive Control	*r*(267) = −.24 [−.39, −.07]	*r*(267) = −.24 [−.39, −.07]	*r*(267) = −.22 [−.38, −.05]
Flanker Rating × Executive Control	*r*(267) = −.10 [−.27, .07]	*r*(267) = −.15 [−.31, .02]	*r*(267) = −.10 [−.27, .07]
SART Rating × Distracted-Restless	*r*(203) = .09 [−.11, .28]	*r*(203) = .19 [−.01, .37]	*r*(203) = .08 [−.12, .27]
Flanker Rating × Distracted-Restless	*r*(203) = .13 [−.07, .32]	*r*(203) = .18 [−.02, .36]	*r*(203) = .11 [−.09, .30]
SART Rating × Pos. Daydreaming	*r*(203) = .02 [−.18, .21]	*r*(203) = .08 [−.12, .27]	*r*(203) = .03 [−.17, .22]
Flanker Rating × Pos. Daydreaming	*r*(203) = .18 [−.02, .36]	*r*(203) = .16 [−.04, .34]	*r*(203) = .17 [−.03, .35]

*TUT* task-unrelated thought, *DSSQ* Dundee Stress State Questionnaire (post-task retrospective report of TUT frequency), *Executive Control* factor scores from a confirmatory factor analysis of four cognitive performance measures (see text for details), *Distracted-Restless* z-score composites of seven questionnaire measures of Distractibility and Restlessness (see text for details), *Pos. Daydreaming* z-score composites of four questionnaire measures of Positive-Constructive Daydreaming (see text for details)

Treating depth ratings as continuous offers no benefit compared to dichotomizing them and using TUT rate. Whether treated as continuous or dichotomous reports (and whether considering depth rating *M* or SD), TUTs correlated strongly positively between SART and flanker tasks, correlated positively with retrospective (post-task) mind-wandering reports, correlated weakly negatively with executive control, and correlated very weakly positively with self-reported behavioral tendencies: One-third of the corresponding *M* depth and dichotomized correlations were identical to two decimal places, and the rest differed by .03 or less; SD depth correlations were slightly weaker than dichotomized correlations in some cases (SART × flanker mind-wandering) and slightly stronger in others (mind-wandering × Distractibility and Restless scores). We therefore conclude that continuous depth reports of on- versus off-task states show no incremental individual-differences validity beyond capturing a broad distinction between on-task versus off-task thought.

Second, taking a within-person approach, we tested whether continuous depth reports predict in-the-moment behaviors in a near-linear fashion, with higher off-task ratings associated with greater behavioral indications of attentional disengagement. That is, if depth ratings accurately reflect subtle gradations in thought focus, we should find evidence for such gradations in behavior. Seli et al. (2014), for example, tested whether TUT depth reports on a 1–5 scale predicted an objective fidgeting measure for trials preceding each thought report. They did not: Only ratings of 5 were associated with more fidgeting than ratings of 1–4. In a classroom context, Wammes and Smilek (2017) asked whether depth reports during lectures predicted quiz performance on material presented near the probes. They found statistical differences in recall only between ratings of 5 versus 2 and 1, and 4 versus 1; reports of 2, 3, and 4 were statistically indistinguishable. Finally, Zanesco et. al. (2020) assessed the association between depth reports (on a 1–6 scale) and the preceding SART trial’s no-go accuracy. They found a quadratic decrease: Accuracy dropped only across ratings of 4–6.

Seli et al. (2014), Laflamme et al. (2018), and Zanesco et. al. (2020) tested in-the-moment associations between depth ratings and *M* RT variability in the computer-task trials preceding each thought report. Here, the statistics suggested linearity, but the patterns were noisy and inconsistent. In each study, some consecutive ratings (e.g., 1–2; 1–3) were associated with no changes in RT variability, and this “flat” part of the distribution changed from sample to sample. Across measures and samples, then, depth reports only sometimes track performance, and even when they do, it is only roughly.

**Fig. 9:**
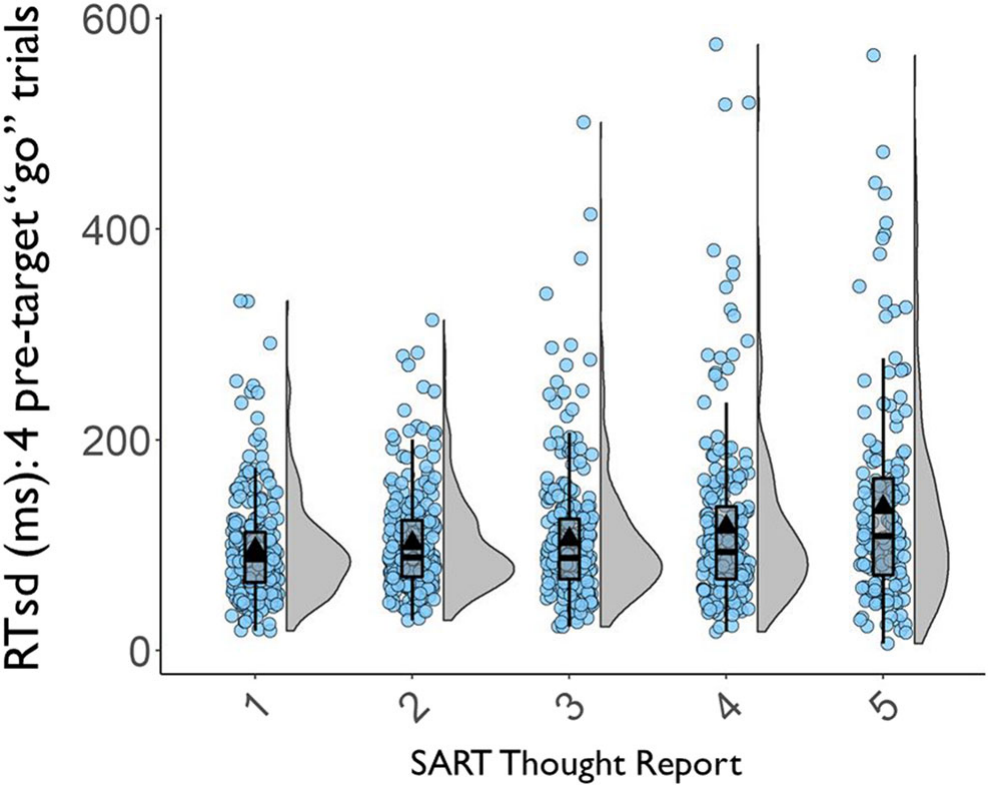
Mean standard deviations in reaction times (RTsd) for the four go-trials preceding each thought report in the SART, for each depth probe response; the depth report response scale was labeled: (1) Completely on-task; (2) Mostly on-task; (3) Both on the task and off-task; (4) Mostly off-task; (5) Completely off-task. Box plots present the 25^th^, 50^th^, and 75% percentiles; whiskers extend to the smallest and largest values within 1.5 times the inter-quartile range. Means are presented as triangles. Each dot represents an individual subject’s *M* RTsd preceding thought reports with that depth rating

We therefore conducted a LMM on RTsd in the SART, for the four go-trials preceding each thought report, as we did in the prior analyses (Fig. 4); here, though, we treated depth reports (1–5) as continuous. Figure 9 suggests only a small increase in median RTsd from rating 4–5 (but not elsewhere), and a slightly greater increase in *M* RTsd from ratings 3–5 (driven by increased skew rather than a shift in the distribution); a linear effect is certainly not obvious. Indeed, relative to depth ratings of 1, *M* RTsd did not increase significantly for ratings of 2, b = 6 ms, SE = 5 ms, *t*(866.6) = 1.14, *p* = .255, or ratings of 3, b = 9 ms, SE = 5 ms, *t*(871.3) = 1.76, *p* = .079, but did increase significantly for ratings of 4, b = 20 ms, SE = 5 ms, *t*(888.4) = 3.75, *p* < .001, and ratings of 5, b = 38 ms, SE = 6 ms, *t*(906.5) = 6.31, *p* < .001; the only significant RTsd increase across consecutive ratings was from 4 to 5, b = 18 ms, SE = 6 ms, *t*(882.1) = 2.92, *p* = .004. RTsd increased only for the most extreme off-task ratings. Thus, despite a very large sample for a within-subjects analysis (*n* = 269), we did not find compelling evidence for a linear association between depth ratings and RTsd, and thus we did not find compelling evidence for the utility or validity of continuous depth reports—either here or in the previously discussed between-person correlational analyses.

### Validity evidence for TUT intentionality reports?

Our primary analyses collapsed TUT rates across reports of intentional and unintentional TUTs in the intentionality (“Why”) condition. Here we explore whether our data provide any evidence for subjects’ validly discriminating the reasons for their TUTs. Our concerns regarding intentionality-report validity come partly from Nisbett and Wilson (1977), who reviewed evidence that subjects frequently misinterpret or confabulate post hoc explanations for their behaviors and experiences; thus, people may err about *why* they mind-wander. Moreover, intentionality probes are ambiguous in referring either to the *initiation* of the thought stream (which may have begun minutes ago, unnoticed and unremembered) or its *maintenance* (which may reflect vacillating intention). Subjects may decide to briefly disengage from an ongoing task but then, much later, be interrupted by a probe that finds them mind-wandering well beyond the intended timeframe; or, subjects may spontaneously slip into TUTs but later, upon noticing, allow that mind wandering to continue. Should they respond *intentional TUT* or *unintentional TUT* to probes under these circumstances?

Despite concerns, the literature provides some empirical-dissociation evidence for the validity of TUT intentionality reports. Experimental manipulations of task contexts show some selective effects: (a) Re-reading a text increased only intentional but not unintentional TUTs versus reading once (Phillips et al., 2016); (b) reading longer passages increased unintentional but not intentional TUTs versus shorter passages (Forrin et al., 2021); (c) Across a task, unintentional TUT rates increased more than intentional (Massar et al., 2020; Robison, 2018), and; (d) A task with unpredictable target-response events yielded higher unintentional than intentional TUT rates (.37 vs. .15) but a task with predictable events yielded similar rates (.27 vs. .23; Seli, Risko, & Smilek, 2016b). Some correlational results also indicate dissociations: (a) Adults with childhood ADD diagnoses reported more unintentional but not intentional TUTs during a vigilance task (Shaw & Giambra, 1993); (b) Working memory capacity correlated more strongly with unintentional than intentional TUT rates (Ju & Lien, 2018; Robison & Unsworth, 2018); (c) Self-reported motivation correlated more strongly with intentional than unintentional TUT rates (Robison & Unsworth, 2018; Seli et al., 2015), and; (d) Intentional TUTs were more strongly associated with future-oriented content than were unintentional TUTs, whereas unintentional TUT were more strongly associated with vague content (Seli, Ralph et al. 2017).

At the same time, some experimental manipulations had statistically identical effects on intentional and unintentional TUT rates, such as cognitive load (Forster & Lavie, 2009), stimulus timing (Unsworth & Robison, 2018), task incentives (Seli et al., 2019), time-on-task (Seli, Ralph et al. 2017), and re-watching videos (Martin et al., 2019). Other studies, moreover, find statistically identical correlates of intentional and intentional TUT rates, including age (Seli, Maillet et al. 2017), self-reported childhood distractibility (Shaw & Giambra, 1993), task performance (Martin et al., 2018; Seli et al., 2015; Seli, Wammes, et al., 2016; Zhang et al., 2020), eye-movements in response to unexpected text events (Zhang et al., 2020), and self-reports of task-related states, such as motivation or interest (Phillips et al., 2016; Robison & Unsworth, 2018).

The interpretative challenge we face, then, is twofold: (1) Not only do different manipulations and individual-differences variables show different dissociative–associative patterns, but theory cannot tell us, *a priori*, where intentional and unintentional TUTs should behave differently versus similarly; (2) When associations or dissociations arise, they may sometimes result, at least in part, from demand characteristics or from folk theories about mind-wandering, motivation, or performance, rather than from a faithful recounting of the initiation or the maintenance (or both?) of the current thought stream. Theory doesn’t tell us when we should or shouldn’t be concerned about demand or folk theory. We will therefore take a relatively comprehensive exploratory approach to characterizing our intentional versus unintentional TUT rates (and their correlates), with the goal of contributing to future efforts at construct validation and theorizing.

#### Characteristics of intentional and unintentional TUT rates

We first simply assess whether intentional and unintentional TUT rates were similar across SART and flanker tasks, and whether they were similarly correlated across tasks. *M* intentional TUT rates were low and dropped modestly, from .16 to .12 across SART and flanker tasks, whereas *M* unintentional TUT rates were higher and dropped substantially, from .32 to .21 across tasks. A 2 (TUT type) × 2 (task) repeated measures ANOVA (jamovi project, 2019)^4^ indicated that unintentional TUTS were more frequent than intentional TUTs, *F*(1, 262) = 78.72, MSE = 0.05, *p* < .001, that TUTs decreased overall across tasks, *F*(1, 262) = 126.90, MSE = 0.01, *p* < .001, and that unintentional TUTs decreased more sharply across tasks than did intentional TUTs, *F*(1, 262) = 22.55, MSE = 0.02, *p* < .001. Paired-sample *t* tests indicated, however, that the cross-task decrease was significant for both intentional TUT rates, *t*(262) = 4.31, *p* < .001, d = 0.27, BF_10_ = 493, and unintentional TUT rates, *t*(262) = 9.85, *p* < .001, d = 0.61, BF_10_ = 3.9 × 10^16^.

Despite different report rates and cross-task change, individual differences in both intentional and unintentional TUTs were stable. SART and flanker intentional TUT rates correlated almost exactly as strongly as unintentional TUT rates: for intentional TUT, *r*(261) = .57 [.44, .68], and for unintentional TUT, *r*(261) = .60 [.48, .70]. Subjects were equally consistent in their propensity for intentional and unintentional TUTs.

Intentional and unintentional TUT reports were made with similar confidence in the SART (*M* confidence ratings = 3.41 and 3.29, respectively), with both lower than on-task reports (*M* = 3.79). Intentional and unintentional TUT reports were made with numerically identical confidence in the flanker task (both *M*s = 4.50), again lower than on-task reports (*M* = 4.89). A 3 (thought report) × 2 (task) repeated measures ANOVA indicated significant differences in confidence across report types, *F*(2, 282) = 27.08, MSE = 0.48, *p* < .001, and tasks, *F*(1, 141) = 24.14, MSE = 0.59, *p* < .001, but no interaction, *F*(2, 282) = 2.73, MSE = 0.21, *p* = .067. Paired-sample *t* tests indicated no differences in confidence between unintentional and intentional TUTs in the SART, *t*(225) = 1.42, *p* = .158, d = .09, BF_10_ = 0.19, or flanker task, *t*(149) = −0.05, *p* = .963, d = −0.00, BF_10_ = 0.09. However, in both tasks, confidence for on-task reports was higher than both intentional and unintentional TUT reports, all *t*s > 6.74, *p*s < .001, ds > 0.43, BF_10_s > 6.2 × 10^7^.

In summary, subjects reported unintentional TUTs more frequently than intentional TUTs. Unintentional TUT rates decreased more across tasks than did intentional TUT rates, but we found few other differences between them: Intentional and unintentional TUT rates were equivalently correlated across tasks and they were made with similar confidence (with both reduced versus on-task reports).

#### Within- and between-subject correlates of intentional and unintentional TUT rates

We focus our correlational analyses on the SART data, where we can examine both within- and between-subject correlates of TUT reports. First, we assess within-subject associations, asking whether intentional and unintentional TUT reports differentially distinguish, in the moment, trials preceded by more versus less RT variation, or error trials from accurate trials. Second, we assess between-subject associations, asking whether intentional and unintentional TUT rates differentially correlate with executive-control performance or our questionnaire-assessed constructs of interest.

##### Within-subject correlates

**Fig. 10:**
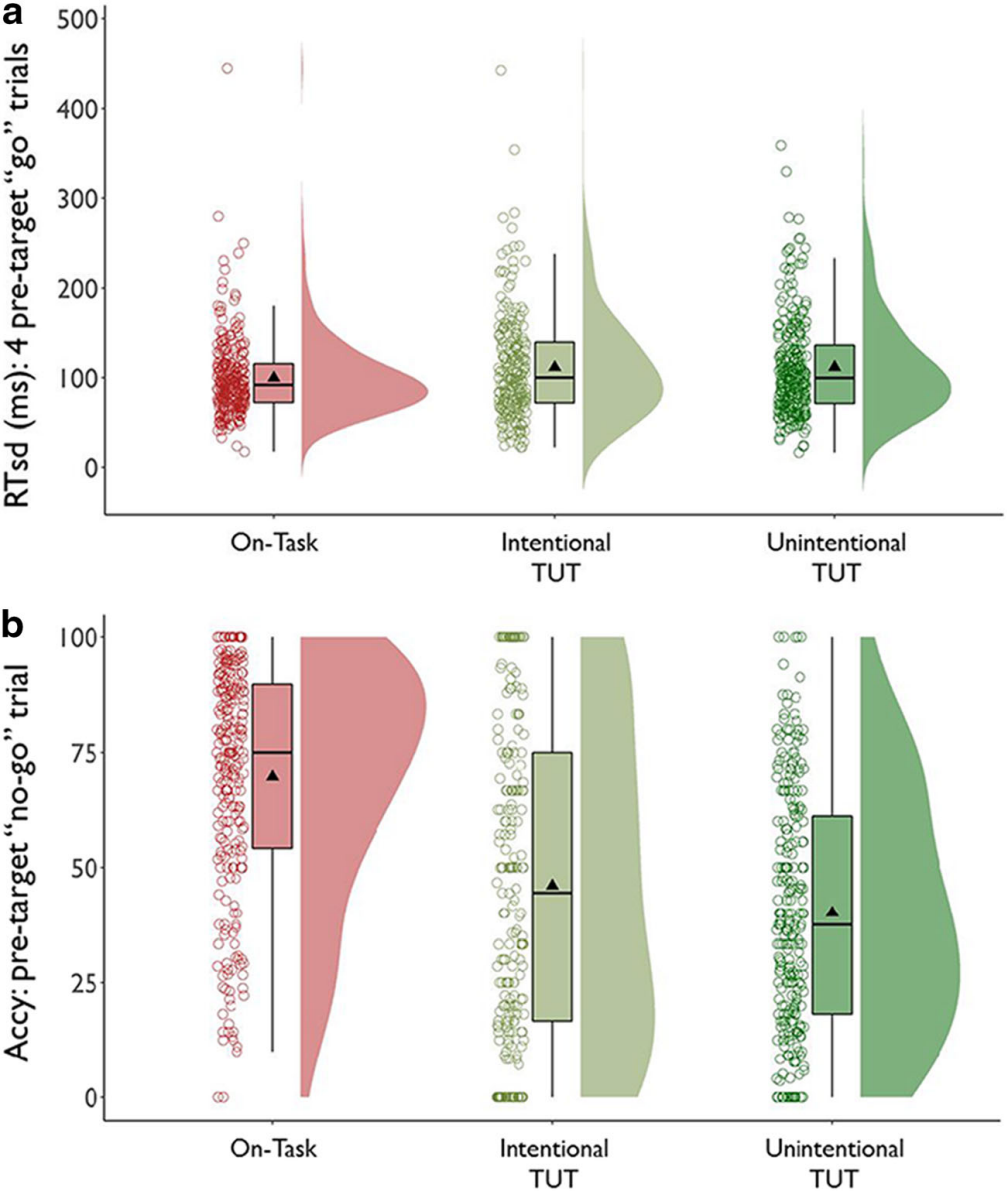
**a** Standard deviations in reaction times (RTsd) for the four go-trials preceding on-task reports, intentional task-unrelated thought (TUT) reports, and unintentional TUT reports in the Sustained Attention to Response Task, for subjects in the intentionality (“Why”) probe condition. Box plot*s* present the 25^th^, 50^th^, and 75% percentiles; whiskers extend to the smallest and largest values within 1.5 times the inter-quartile range. Means are presented as triangles. Each dot represents an individual subject’s RTsd preceding on-task, intentional TUT, or unintentional TUT reports. **b** Accuracy (“Accy”) rates for no-go trials preceding on-task reports, intentional task-unrelated thought (TUT) reports, and unintentional TUT reports in the Sustained Attention to Response Task, for subjects in the intentionality (“Why”) probe condition. Box plots present the 25th, 50th, and 75% percentiles; whiskers extend to the smallest and largest values within 1.5 times the inter-quartile range. Means are presented as triangles. Each dot represents an individual subject’s accuracy rate preceding on-task, intentional TUT, or unintentional TUT reports

We first examined RTsd across the four “go” trials preceding each no-go trial and its thought report. As shown in Fig. 10a, subjects responded more variably prior to both intentional and unintentional TUTs relative to on-task thought. LMM contrasts indicated that RTsd was modestly but significantly greater preceding intentional TUTs than on-task reports, b = 12 ms, SE = 3 ms, *t*(6869) = 3.77, *p* < .001, and preceding unintentional TUTs than on-task reports, b = 11 ms, SE = 3 ms, *t*(6862) = 4.40, *p* < .001; RTsd preceding intentional versus unintentional TUT reports, however, did not differ significantly, b = 1 ms, SE = 3 ms, *t*(6869) = 0.28, *p* = .778. We next examined no-go accuracy preceding each report. Figure 10b indicates that subjects were more likely to commit an error prior to TUT versus on-task reports, but this difference was larger for unintentional than intentional TUTs. GLMM contrasts indicated that, compared to on-task reports, accuracy was lower preceding intentional TUTs, b = −1.30, SE = .07, Z = −19.45, *p* < .001, and unintentional TUTs, b = −1.60, SE = .05, Z = −29.33, *p* < .001; moreover, accuracy was lower preceding unintentional than intentional TUTs, b = −0.30, SE = .07, Z = −4.49, *p* < .001. Whereas RTsd showed similar effects across thought reports, accuracy did not, suggesting that reactivity to response accuracy may have colored unintentional versus intentional TUT reports.

##### Between-subject correlates

Table 5 presents correlations for intentional and unintentional SART TUT rates (and proportion of TUTs that were intentional, for subjects with ≥ 1 TUT report), with executive-control scores, post-SART DSSQ mind-wandering scores, and questionnaire composites for distractibility–restlessness and positive-constructive daydreaming. The proportion of subjects’ TUTs that were intentional did not correlate with any constructs of interest. Unintentional TUT rates correlated numerically more strongly than did intentional TUT rates with DSSQ, executive control, and distractibility–restlessness, with the latter correlation being statistically significant for only unintentional TUTs rate (and only these distractibility-restlessness correlations differed beyond the ±.10 stability corridor for intentional versus unintentional TUTs). None of the differences in correlation magnitude were statistically significant (by Williams test of dependent correlations; for the latter distractibility-restlessness correlations, *t*[198]= 1.37, *p* =.171).

**Table 5:** Correlations (with 99.5% confidence intervals) for TUT rates from subjects in the intentionality (“Why”) condition, for intentional TUT rate, unintentional TUT rate, and proportion of all TUTs that were intentional, in the SART

Outcome variable	Intentional TUT rate	Unintentional TUT rate	Prop (Intent TUT / Total TUT)
SART DSSQ	*r*(219) = .23 [.04, .40]	*r*(219) = .35 [.17, .50]	*r*(213) = .00 [−.19, .19]
Executive Control	*r*(261) = −.08 [−.25, .09]	*r*(261) = −.13 [−.30, .04]	*r*(255) = .02 [−.15, .19]
Distractibility-Restlessness	*r*(199) = .09 [−.11, .28]	*r*(199) = .23 [.03, .41]	*r*(194) = −.01 [−.21, .19]
Pos.-Constructive Daydream	*r*(199) = .22 [.02, .40]	*r*(199) = .18 [−.02, .36]	*r*(194) = .09 [−.11, .28]

*TUT* task-unrelated thought, *SART* Sustained Attention to Response Task, Prop (Intent TUT / Total TUT) = proportion of a subject’s TUT reports that were intentional TUTs, *DSSQ* Dundee Stress State Questionnaire (post-task retrospective report of TUT frequency); Executive Control = factor scores from a confirmatory factor analysis of four cognitive performance measures (see text for details); Distractibility-Restlessness = z-score composites of seven questionnaire measures of Distractibility and Restlessness (see text for details); Pos.-Constructive Daydream = z-score composites of four questionnaire measures of Positive-Constructive Daydreaming (see text for details)

In summary, intentional and unintentional TUTs were equivalently associated, in the moment, with preceding RT variability. They were also equivalently associated (or unassociated) with individual differences in executive control and our self-report constructs of interest. Both intentional and unintentional TUT reports were more strongly associated, in the moment, with no-go errors than were on-task reports, but unintentional TUTs were more strongly associated with these errors than were intentional TUTs.

### Consequences of not probing for TRI content

Our final analyses address probes assessing thought content (“*What*”) that do versus don’t provide a TRI reporting option. Subjects frequently endorse TRI when provided as a response option (e.g., Jordano & Touron, 2017; Mrazek et al., 2011), but most studies’ probes don’t do so. Only Robison et al. (2019) directly assessed the consequences of not probing for TRI. Three groups of subjects completed the SART; each probe asked them to endorse either: (a) on-task thought or TUT (two choices; *n* = 29); (b) on-task thought, TUT, or TRI (three choices; *n* = 30), or; (c) on-task thought, TUT, TRI, external distractions, or mind-blanking (five choices; *n* = 29). Of most relevance here, subjects in the three-choice condition reported TRI to 29% of probes; compared to the no-TRI condition, their on-task reports dropped by 20% and TUTs by 10%, suggesting that most TRI experiences in the no-TRI condition were reported as on-task, but some were reported as TUT.^5^

The two- versus three-choice comparisons suggest two-thirds of TRI experiences will be characterized as on-task and one-third as TUT when TRI reporting is prevented. We address the influence of TRI probing on thought-report validity by contrasting the findings from our content-probes conditions that did (*n* = 266) versus didn’t (*n* = 269) provide a TRI reporting option.

#### Rates of TRI, TUT, and on-task reports

**Fig. 11:**
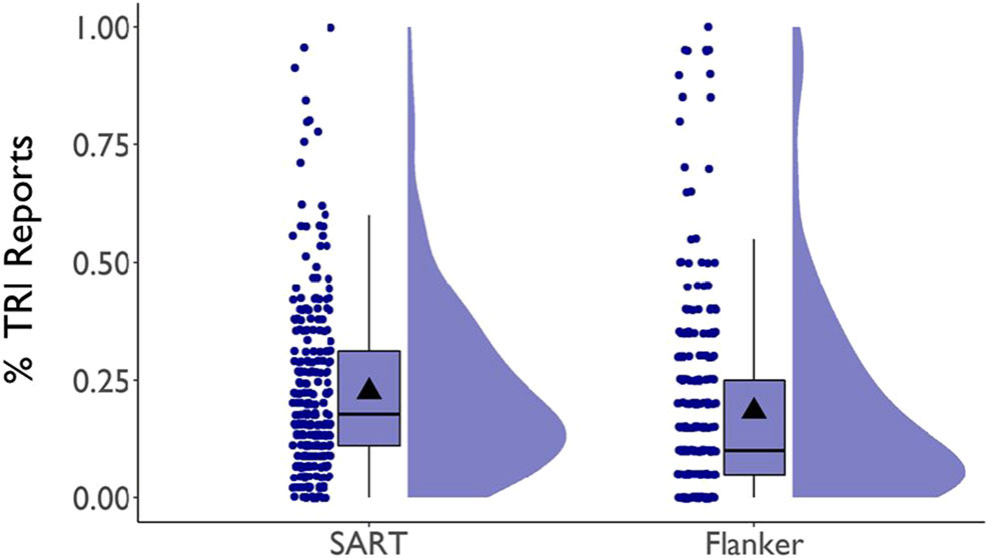
Proportion of thought probes yielding task-related interference (TRI) reports in the Sustained Attention to Response Task (SART) and the flanker task, for subjects in the content (“What”) probe condition including TRI as a response option. Box plots present the 25^th^, 50^th^, and 75% percentiles*; *whiskers extend to the smallest and largest values within 1.5 times the inter-quartile range. Means are presented as triangles

Figure 11 presents slightly decreasing TRI rates from the SART (*M* proportion = .23) to the flanker task (*M* proportion = .18), for subjects seeing content probes with a TRI option. This decrease was not significant, however, *t*(524.5) = 2.42, *p* = .016, d = 0.21 [95% CI = 0.04, 0.38], with weak evidence for the alternative model over the null, BF_10_ = 1.63. Subjects reported TRI at about 20% of probes, then, across tasks; the SART TRI mean (.23) here is reasonably consistent with the estimate (.29) from Robison et al. (2019).

**Fig. 12:**
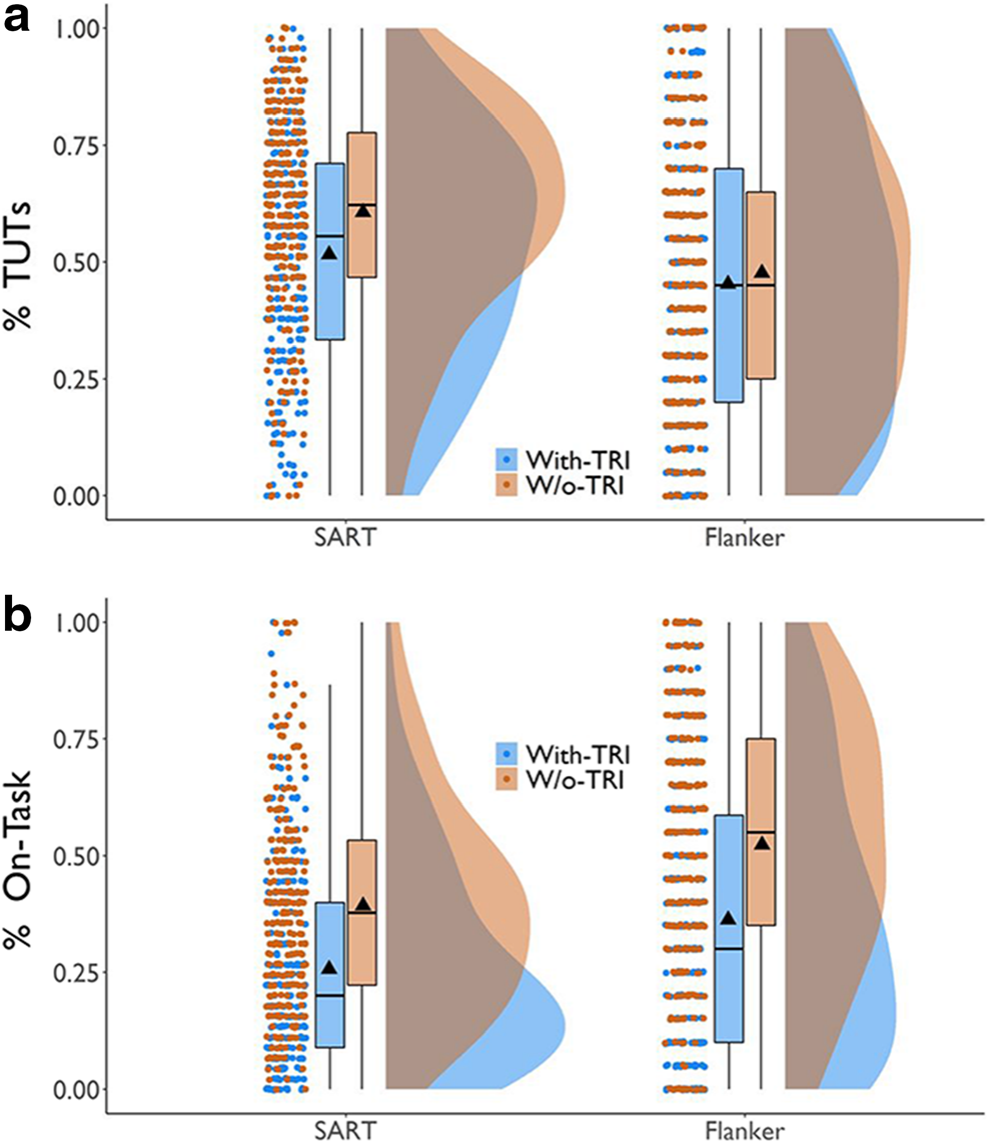
**a** Proportion of task-unrelated thoughts (TUTs) reported in the Sustained Attention to Response Task (SART) and the flanker task, for subjects in the content probe condition with task-related interference (TRI) as a response option versus the content probe condition without (W/o) TRI as a response option. Box plots present the 25^th^, 50^th^, and 75% percentiles; whiskers extend to the smallest and largest values within 1.5 times the inter-quartile range. Means are presented as triangles. **b** Proportion of on-task thoughts reported in the Sustained Attention to Response Task (SART) and the flanker task, for subjects in the content probe condition with task-related interference (TRI) as a response option versus the content probe condition without (W/o) TRI as a response option. Box plots present the 25^th^, 50^th^, and 75% percentiles; whiskers extend to the smallest and largest values within 1.5 times the inter-quartile range. Means are presented as triangles

We next consider how manipulating the TRI reporting option may have affected TUT reports. Figure 12a presents TUT rates in both the tasks, for subjects responding to probes with versus without a TRI option. In the SART, subjects without a TRI option reported more TUTs than did those with a TRI option, but in the flanker task the TUT rates were more similar. The ANOVA table indicated the TUT rates were higher for subjects without versus with a TRI reporting option, *F*(1, 533) = 8.06, *p* = .005, but this probe-condition effect interacted with task, *F*(1, 533) = 10.50, *p* = .001. A LMM (with the reference level set to the flanker task in the TRI-option condition) indicated that, in the flanker task, TUT rates did not differ significantly between probe-type conditions (*M*s = .47 vs. .45), b = 0.02, SE = 0.02, *t*(796.0) = 1.04, *p* = .297. For the TRI-option condition, TUT rate was significantly higher in the SART than in the flanker task (*M*s = .51 vs .45), b = 0.06, SE = 0.01, *t*(533.0) = 4.34, *p* < .001, and TUT rate increased still further in the SART for the No-TRI-option condition (*M* = .58), b = .07, SE = .02, *t*(533.0) = 3.24, *p* = .001. Thus, in the initial SART task, denying subjects a way to report TRI increased their TUT reports modestly, suggesting that at least some TRI experiences were initially reported as TUTs, but this tendency disappeared in the second, flanker task.

Figure 12b illustrates on-task thought reports. Here, in both tasks, subjects without a TRI option reported more on-task experiences than did those with a TRI reporting option; these probe-condition differences appeared larger than those in TUTs. The ANOVA table indicated that on-task rates were higher in the No-TRI than the TRI-option condition, *F*(1, 533) = 58.48, *p* < .001, and no interaction with task, *F*(1, 533) = 1.51, *p* = .220. A LMM indicated that, in the flanker task, on-task reports were more frequent in the No-TRI than the TRI condition (*M*s = .52 vs. .36), b = .16, SE = .02, *t*(791.0) = 7.37, *p* < .001; moreover, the significant difference in on-task rates from the SART to the flanker task in the TRI-option condition (*M*s = .25 vs. .36), b = −.11, SE = .01, *t*(533.0) = −7.59, *p* < .001, was statistically equivalent to that in the no-TRI condition, b = −.02, SE = .02, *t*(533.0) = −1.23, *p* = .220.

When TRI is not provided as a response option, these experiences are sometimes reported as TUT, but more often as on-task thought (see Robison et al., 2019). Thus, analyses aimed at mean TUT rates won’t be affected much by the exclusion of TRI reporting, whereas analyses aimed at on-task thought—or differences between TUT and on-task thought—will be greatly affected.

#### Correlates of TUT rates

Although the preceding analyses suggested that TUT rates change only modestly with the exclusion of the TRI reporting option, here we examined whether individual differences in TUT rates varied across the content-probing conditions that did versus didn’t provide a TRI response option. In short, they did not. As shown in Table 6, there were no statistically significant differences in SART and flanker TUT rate correlations with each other, or in either’s correlation with DSSQ TUT ratings, executive-control abilities, or retrospective ratings of propensity for distractibility and restlessness or for positive-constructive daydreaming. The only numerically notable (but non-significant) differences between the TRI-report-option condition and the no-TRI condition were for SART TUT rate × executive control (*r*s = −.32 vs. −.20, where both were significant), flanker TUT rate × executive control (*r*s = −.22 vs. −.06, where only the former was significant), and flanker TUT rate × distractibility and restlessness (*r*s = .21 vs. .06, where only the former was significant). We therefore find no statistical evidence for TRI inclusion affecting TUT-rate correlations, but future replication work with larger subject samples might profitably target these suggestive patterns.

**Table 6:** Correlations (with 99.5% confidence intervals) for TUT rates with other outcomes of interest, from subjects in the Content probe condition with a TRI option and from subjects in the Content probe condition without a TRI option, with statistical tests for differences in independent correlations

Correlation	Content probes with TRI	Content probes without TRI	Difference test
SART TUT × Flanker TUT	*r*(264) = .61 [.49, .71]	*r*(267) = .57 [.44, .67]	z = 0.71, *p* = .478
SART TUT × SART DSSQ	*r*(225) = .32 [.14, .48]	*r*(223) = .27 [.09, .43]	z = 0.58, *p* = .562
Flanker TUT × Flanker DSSQ	*r*(225) = .40 [.23, .54]	*r*(223) = .32 [.14, .48]	z = 0.98, *p* = .327
SART TUT × Executive Control	*r*(264) = −.32 [−.47, −.16]	*r*(267) = −.20 [−.35, −.03]	z = 1.56, *p* = .119
Flanker TUT × Executive Control	*r*(264) = −.22 [−.38, −.05]	*r*(267) = −.06 [−.23, .11]	z = 1.95, *p* = .051
SART TUT × Distract-Restless	*r*(205) = .06 [−.13, .25]	*r*(207) = .04 [−.15, .23]	z = 0.23, *p* = .818
Flanker TUT × Distract-Restless	*r*(205) = .21 [.02, .39]	*r*(207) = .06 [−.13, .25]	z = 1.59, *p* = .112
SART TUT × Pos.-Constructive	*r*(205) = .06 [−.13, .25]	*r*(207) = .07 [−.12, .26]	z = 0.07, *p* = .944
Flanker TUT × Pos.-Constructive	*r*(205) = .18 [−.01, .36]	*r*(207) = .10 [−.09, .29]	z = 0.80, *p* = .424

*TUT* task-unrelated thought, *TRI* task-related interference, *SART* Sustained Attention to Response Task, *DSSQ* Dundee Stress State Questionnaire (post-task retrospective report of TUT frequency); Executive Control = factor scores from a confirmatory factor analysis of 4 cognitive performance measures (see text for details); Distract-Restless = z-score composites of 7 questionnaire measures of Distractibility and Restlessness (see text for details); Pos.-Constructive = z-score composites of 4 questionnaire measures of Positive-Constructive Daydreaming (see text for details)

#### Correlates of TRI rates

Finally, we examine how TRI rates correlate with other constructs (see Table 7). Individual differences in TRI were consistent across SART and flanker tasks. As well, TRI rates correlated with post-task DSSQ TRI ratings (in the .25 range) similarly to the corresponding TUT–DSSQ correlations (in the .30–.35 range). Although TRI rates in the SART correlated significantly positively with executive-control performance, TRI in the flanker task did not, apparently reflecting the mixed findings in the literature of positive or null associations (McVay & Kane, 2012a; Robison & Unsworth, 2018; Stawarczyk et al., 2011; Unsworth & McMillan, 2017; Welhaf et al., 2020). As might be expected, TRI rates did not correlate significantly with our retrospective questionnaires of distractibility or constructive daydreaming propensities.

**Table 7:** Correlations (with 99.5% confidence intervals) for TRI rates with other outcomes of interest, from subjects in the Content probe condition with a TRI option

SART TRI × Flanker TRI	*r*(264) = .60 [.48, .70]
SART TRI × SART DSSQ (TRI scale)	*r*(225) = .26 [.08, .42]
Flanker TRI × Flanker DSSQ (TRI scale)	*r*(225) = .24 [.06, .41]
SART TRI × Executive Control	*r*(264) = .18 [.01, .34]
Flanker TRI × Executive Control	*r*(264) = .07 [−.10, .24]
SART TRI × Distract-Restless	*r*(205) = .03 [−.17, .22]
Flanker TRI × Distract-Restless	*r*(205) = −.17 [−.35, .02]
SART TRI × Pos.-Constructive	*r*(205) = .00 [−.19, .20]
Flanker TRI × Pos.-Constructive	*r*(205) = −.10 [−.29, .10]

*TRI* task-related interference, *SART* Sustained Attention to Response Task, *DSSQ* Dundee Stress State Questionnaire (post-task retrospective report of TRI frequency); Executive Control = factor scores from a confirmatory factor analysis of four cognitive performance measures (see text for details); Distract-Restless = z-score composites of seven questionnaire measures of Distractibility and Restlessness (see text for details); Pos.-Constructive = z-score composites of four questionnaire measures of Positive-Constructive Daydreaming (see text for details)

## General discussion

Despite hundreds of published articles on mind wandering, we identified fewer than a dozen studies *ever* conducted to explicitly validate probed TUT rates. The construct validity of TUT reports can, of course, be inferred from other, more theoretically oriented studies in the literature; our introduction reviewed some of the most compelling positive evidence. However, the lack of systematic validation work identified by Weinstein’s (2018) critique of the field indicates that, like much of psychology (Borsboom, 2006; Flake & Fried, 2020), mind-wandering research has not adequately addressed measurement.

The present study explored the construct validity of probed TUT rates with a combined experimental and individual-differences approach. We examined TUT reports from over 1000 undergraduates at two U.S. institutions, who responded to one of four different thought-probe types across two tasks. We asked a fundamental measurement question: Do different probe types yield different results, either in terms of average reports (average TUT rates, mean confidence ratings for TUT reports) or TUT-report *associations*, such as between-task TUT rates or confidence ratings, or between TUT reports and other consciousness-related constructs (e.g., executive-control performance, self-reports of positive-constructive daydreaming)?

Our primary analyses compared probes that asked subjects to report on different dimensions of experience: *what* they’d been mind-wandering about, *why* they were mind-wandering, or the *extent* of their mind-wandering. Our secondary analyses compared thought-content probes that did versus didn’t allow TRI reports. Our findings provide both some “good news”—that some findings are robust across probing methods—and some “bad news”—that some findings may not be robust across methods and that some commonly used probing methods may not tell us what we think they do.

### The good news

Supporting the literature’s measurement practices and assumptions, many results did not differ by probe type. In primary analyses, all probe conditions yielded nearly identical TUT rates in the SART, as well as significantly lower TUT rates in the flanker task. (Although the depth-rating cut-off for defining TUTs was made to approximate the content- and intentionality-condition TUT rates, the latter two varied freely, and the depth cut-off yielded consistent results with a daily-life study [Seli, Beaty, et al., 2018]). Self-reported confidence in TUT reports increased from the SART to flanker tasks for all probe types. Individual differences in TUT rates and TUT confidence ratings were equivalently reliable across tasks for all probe types. Regarding within-person associations, all probe types showed modest-but-significant increases in RT variability on SART trials preceding TUT versus on-task reports, and large decreases no-go accuracy on trials preceding TUT versus on-task reports. Regarding between-person associations, TUT rates for all probe types correlated statistically equivalently with post-task retrospective ratings of TUT experiences in both tasks, with objective executive-control factor scores, and with composite self-report trait measures of distractibility–restlessness and positive-constructive daydreaming.

In secondary analyses about TRI-inclusive probes, TUT rates dropped from the SART to flanker tasks for probes either including or excluding a TRI-report option, and TUT rates were equivalently reliable across tasks for both probe types. TUT rates from both probe types were equivalently correlated with post-task retrospective mind-wandering ratings for both tasks, with executive-control scores, with self-reported distractibility–restlessness, and with self-reported propensity for positive-constructive daydreaming.

### The bad news

Despite many TUT-rate similarities across probe types, we also found differences that complicate the interpretation of probed TUT reports.

#### Differences across probe types

The drop in mean TUT rate from SART to flanker was twice as steep for the intentionality and depth probes than for content probes. The resulting TUT rate in the flanker task, moreover, was significantly higher for the content-probe condition than for the intentionality and depth conditions (with a *M* proportion difference of over .10). Our design, which presented SART and flanker tasks in a fixed order, does not allow a clear explanation; the poorer TUT-rate stability across tasks for intentionality and depth probes may have been caused by processes associated with practice, general fatigue, or something specific to the flanker task.

Confidence ratings in TUT reports were also significantly higher (and more similar to on-task confidence) in the content than in the other probe conditions, in both tasks: *M* confidence ratings for content versus depth TUT reports differed by about a half point on a five-point scale. Subjects indicated more certainty when reporting the content of their TUTs than when reporting their intentionality or depth.

Within-person associations also suggested differential validity: Whereas all probe conditions elicited similarly small RTsd increases preceding TUT reports versus on-task reports, they diverged dramatically in the no-go accuracy rates preceding TUT reports versus on-task reports. For all probe types, no-go accuracy was higher preceding on-task than TUT reports, but this accuracy difference in the intentionality- and depth-probe conditions (27 and 29%, respectively) dwarfed that in the content condition (11%). Our joint findings, of no measurable condition effect on RTsd, but a large effect on accuracy, may indicate differential susceptibility to performance-reactive effects across probe types. That is, RTsd is not likely available to introspection: It increased by less than 10 ms, on average, preceding TUT reports. Subjects probably didn’t use perceptions of their RTsd to inform their thought reports. No-go errors, in contrast, are often obvious to subjects (eliciting an audible “*oh, no!*” or more colorful exclamation) and so might influence subsequent thought reports (Head & Helton, 2018). When the obvious and introspectable outcome differed by probe-type, but the non-obvious and non-introspectable one didn’t, we suspect that TUT reports made to intentionality and depth probes are more vulnerable to bias from preceding task performance than are those made to content probes.

#### Problems with depth-rating probes

We see further bad news in our targeted exploration of depth-probe rating scales. Prior studies (e.g., Christoff et al., 2009; Mrazek et al., 2012; Seli, Beaty, et al., 2018) have assumed that mind wandering is a graded experience along a continuum from on- to off-task thinking, and that people can accurately report on this graded experience. Instead, they should have treated both assumptions as requiring theoretical justification and as hypotheses for rigorous testing.

Tay and Jebb (2018) developed a relevant approach to validating continuous constructs. When theorists propose an on-task–off-task continuum, they should first ask whether it is quantitative. If yes (or in exploring whether the answer might be yes), they must define the poles’ meanings, that is, “completely” on-task and “completely” off-task thought: *What should these extreme subjective experiences entail?* Next—and more challenging—theorists must specify the intermediate gradation of subjective experiences between the poles (e.g., via construct mapping; M. R. Wilson, 2005): *What aspects of consciousness should change between being “completely” versus “mostly” on-task, and between “completely” versus “mostly” off-task? By how much? How should experiences of “mostly on-task” or “mostly off-task” differ from each other and from “both on the task and off-task?”* Operationalization questions also necessarily arise, of how many response options amply divide the experience continuum, how the scale points should be labeled, and how they should be explained to subjects. The final outstanding problem, of course, is determining how subjects generate their numerical responses to the scale at each probe and whether all (or most, or some) subjects do so via the same processes.^6^

Regarding the latter problem of response generation, we replicated the finding from a daily-life study (Seli, Beaty, et al., 2018) that subjects given 1–5 depth-probe scales endorsed responses from the midpoint to the “off-task” pole (ratings 3–5) at the same average rate that subjects given category probes endorsed TUTs. Following prior studies, our scale midpoint was confounded with “off-task” labeling (applied to points 3–5), so we don’t know whether the midpoint, the label, or both, drove subjects to use the scale midpoint as the criterion for a “TUT” experience. Such ambiguity about response criteria is a broad problem for the field because in the absence of preregistration, depth probes allow researchers post hoc degrees of freedom in defining TUTs, which could hinge upon the most favorable results. Considerable theoretical and empirical work is needed on the construct validation of depth-probed TUT reports.

Unfortunately, our findings question the value of such theoretical and empirical work. First, relative to content probes, depth-probed TUT rates were: (a) less consistent across tasks; (b) made with less confidence, and; (c) seemingly more reactive to performance. Second, by assessing subjects’ confidence in each thought-probe response, we found troubling patterns suggesting that many subjects confounded their depth reports with degree of confidence. Third, concerns arise from our correlational findings. Like most studies examining within-person associations between depth reports and graded dimensions of in-the-moment behavior (Laflamme et al., 2018; Seli et al., 2014; Wammes & Smilek, 2017), we failed to find a convincingly linear association between depth ratings and SART RT variability over preceding trials. Depth ratings did not track performance as an underlying continuum would imply. Regarding between-person variation, treating depth reports (whether their *M* or their SD) as a continuous variable did not improve correlations with any other constructs compared to deriving a TUT rate from dichotomized depth reports. Not only do depth-probed thought reports have questionable construct validity, then, but they also show no benefit for predictive validity.

Only one newly published study (Zanesco et al., 2020) has critically examined TUT depth-probe ratings. Analyzing sequences of ratings (on a 1–6 scale) across consecutive SART probes, they found different transition patterns for different ratings, with many being asymmetrical. For example, on-task ratings of 1 repeated more frequently across consecutive probes (84% of the time) than did off-task ratings of 6 (64%), and ratings of 6 transitioned to ratings of 1 (19%) more often than the reverse (2%); whereas ratings of 3 were more likely to transition to 2 (25%) than to cross the “off-task” line to 4 (11%), ratings of 4 were less likely to transition to 5 (11%) than to cross the “on-task” line to 3 (19%).

These descriptive findings suggest that all scale points and intervals were not equivalent in subjects’ minds. Moreover, of most theoretical importance, Markov-chain modeling of these transition probabilities suggested ratings were driven by three distinct underlying (hidden) states rather than a graded on-task/off-task continuum or a different state for each depth rating. State 1 was most frequent (at 66% of all probes; in 88% of subjects’ data) and was characterized by almost exclusive ratings of 1 (91% of the time) and some of 2 (6%), lasting an average duration of 18 probes. State 2 was next most frequent (at 25% of all probes; in 48% of subjects’ data) and was characterized by a broad mix of ratings of 1 (14%), 2 (42%), 3 (28%), and 4 (12%), lasting 13 probes. State 3 was least frequent (8% of all probes; in 20% of subjects’ data) and was characterized mostly by ratings of 6 (60%) and 5 (19%), lasting 10 probes. These findings (broadly replicated in two independent datasets) seem to suggest that depth probes may be useful methods to derive estimates of discrete underlying attentional states—rather than an on–off-task continuum. However, we don’t know whether these patterns are influenced by the confidence confounds we observed in our data, and it’s not yet clear how to interrogate the psychological processes that cause shifting among ratings within states (e.g., of 1–4 within state 2) or whether the considerable individual differences in these state profiles are reliable and psychologically meaningful.

It has been more than 10 years since Christoff et al. (2009) introduced depth-probe reports to the mind-wandering literature. The subsequent dearth of serious theorizing and operationalization (à la Tay & Jebb, 2018), or compelling empirical evidence for validity, lead us to discourage the casual use of continuous depth-probe scales in mind-wandering research, at least until such validation work is available.

#### Problems with retrospective mind-wandering reports

Many studies of mind wandering use retrospective questionnaire assessments; some questionnaires are taken immediately after a task and ask about experiences during the task (such as the DSSQ; Matthews et al., 1999), and others ask about more general tendencies toward off-task thought (e.g., Brown & Ryan, 2003; Carriere et al., 2013; Mowlem et al., 2019; Mrazek et al., 2013; Singer & Antrobus, 1970). These questionnaires allow efficient data collection, but their validity as measures of individual differences in mind-wandering propensity rests on people’s ability to notice their fleeting conscious experiences as they occur, to faithfully recall and aggregate them over long (typically unspecified) timescales, and then to accurately translate that aggregation into a relative frequency or agreement rating. We have concerns.

We asked how well probed TUT reports correlated with post-task DSSQ TUT ratings and retrospective-questionnaire scores. Our findings aren’t encouraging. First, for content-, intentionality-, and depth-probe conditions, TUT rates elicited from the SART and flanker tasks correlated more strongly with each other (*r*s ≈ .63), *across tasks*, than SART TUT rates correlated with SART DSSQ TUT ratings (*r*s ≈ .37), or flanker TUT rates correlated with flanker DSSQ TUT ratings (*r*s ≈ .37), *within tasks*. Although TUT–DSSQ correlations approaching .40 aren’t trivial, these two ostensible indicators of identical experiences during the exact same activity shared less than 15% of their variance.

When questionnaires given immediately after tasks show modest correlations with probed TUT rates during those tasks, retrospective questionnaires asking about even more general propensities should show still weaker associations. Three scales from our battery focused on prototypical mind-wandering experiences, the IPI Daydreaming and Mind Wandering scales (Singer & Antrobus, 1970) and the MWS Spontaneous Mind Wandering scale (Carriere et al., 2013). Collapsed across probe-type conditions, the questionnaires correlated strongly with each another (*r*s = .58–.67). They correlated only weakly, however, with TUT rate from the SART (*r*s = .11–.14) and flanker tasks (*r*s = .14–.21). Indeed, prior research bears out weak-to-moderate correlations between probed TUT rates and various mind-wandering questionnaires, ranging from .21–.35, with most less than .30 (Mrazek et al., 2013; Seli, Risko, & Smilek, 2016a; Smeekens & Kane, 2016). Given all the challenges to eliciting valid reports of *immediate* conscious experience (e.g., Hurlburt, 2011), we have little confidence in retrospective reports of mind wandering.

#### Inconsistencies regarding TRI probing

Replicating Robison et al. (2019), subjects’ on-task report rates were greatly reduced for those responding to probes with a TRI option versus those without (−14% in the SART; −16% in the flanker task). These declines suggest that, without a TRI reporting option, subjects frequently report TRI experiences as on-task. In contrast, subjects’ TUT rates fell significantly only in the SART (−7%) but not in the flanker task (−2%), indicating that only in the SART were a meaningful (but still small) proportion of TRI experiences reported as TUTs. Together, our findings indicate that excluding TRI options from probes may modestly bias TUT rates, but it will more strongly bias on-task rates, suggesting some “good news”: Studies that only examine TUTs and are not concerned about on-task rates may exclude TRI reporting options without a major threat to validity (although our SART data suggest some bias is possible). We speculate that such TUT-focused studies without TRI reporting might reduce any validity threats further by explicitly describing to subjects that TRI experiences should be classified as on-task thoughts, to get all subjects on the same page.

### Remaining ambiguities and challenges

Several of our “good news” findings of similarities across probe types, particularly those comparing TUT correlations, rested on non-significant *p* values; given our conservative alpha level of .005, we suggest that some of these statistical non-effects reflected large enough effect sizes to warrant follow-up investigation.^7^ After discussing these, we will turn our attention to the challenges in evaluating the construct validity of probed TUT-intentionality reports.

#### Null effects

Correlations between executive-control scores and TUT rates from the SART and flanker tasks were statistically equivalent across probe conditions. Yet, whereas both correlations were significant for the content condition (*r*s = −.32 and −.22, respectively), they were weak and non-significant for the intentionality condition (*r*s = −.16 and −.04, respectively) and for the depth condition in the flanker task (*r* = −.10). Unfortunately, corresponding BFs did not consistently indicate evidence favoring either the null or alternative hypothesis (only the −.04 correlation yielded BF_10_ < .30). Researchers interested in how to best assess the association between executive control and TUTs cannot yet be sure, then, whether intentionality or depth probes provide less valid assessments of executive-related variation than do content probes.

Just as content-probed TUTs were nominally (but not statistically) better correlated with executive control, intentionality-probed TUTs were nominally (not statistically) better correlated with retrospective reports of everyday distractibility–restlessness and positive-constructive daydreaming. In fact, only intentionality-probed TUT correlations were significant for both everyday constructs in both tasks. Correlations from the content and depth conditions were mostly non-significant, and although some BFs provided modest evidence in favor of the null, others did not. Researchers interested in how intentional TUTs contribute to assessments of individual differences in mind wandering cannot yet be sure, then, whether responses to intentionality probes have more in common with subjects’ general beliefs about their distractibility and related experiences than do responses to content or depth probes.

We therefore have only suggestive (but not statistical) evidence that: (a) content-probed TUTs are more strongly associated with executive control than are intentionality- or depth-probed TUTs, and; (b) intentionality-probed TUTs are more strongly associated with retrospective ratings of negative and positive mind wandering than are content- or depth-probed TUTs. Targeted replication work with large samples—perhaps with preregistered equivalence tests to allow statistical claims for non-differences—is therefore needed to draw provisional conclusions about whether different probe types differentially capture abilities, vulnerabilities, and experiences related to TUT rate.

Such replications are important. First, researchers pursuing executive-control contributions to mind wandering should know whether content probes are both less vulnerable to performance reactivity and more sensitive to executive-related variation, than are intentionality or depth probes. Second, researchers pursuing the influence of intentionality on mind wandering should know whether intentionality probes yield TUT rates that are more tainted by performance reactivity and prior beliefs than do content probes, given the possibility that intentionality-probed TUT reports not only vary more with prior performance accuracy, but also that intentionality-probed TUT rates may covary more with general reports of distraction and daydreaming.

#### Assessing intentionality

We had several *a priori* concerns about probed reports of TUT intentionality: (a) They may be especially vulnerable to confabulation and bias from folk theory and personal beliefs; (b) They may confound reports of TUT initiation with maintenance, both of which are vulnerable to memory errors; (c) Probed rates of intentional and unintentional TUTs have produced mixed findings of dissociations and non-dissociations; (d) Theories of mind wandering and of intentionality are too underspecified to indicate where intentional and unintentional TUT reports should diverge versus converge and what behavioral markers would (in)validate them.

Our exploration of intentional and unintentional TUTs found both similarities and differences, but due to the limitations mentioned above it is hard to know what to conclude from either. Rates of both were strongly and similarly correlated between SART and flanker tasks (*r*s ≈ .60). Both yielded similar levels of reporting confidence. Both were preceded by similar levels of RT variability. Both were similarly correlated with retrospective DSSQ TUT ratings (significantly), executive control (non-significantly), and positive-constructive daydreaming (significantly for only intentional TUT rate, but with similar effect sizes). The significant differences we found between intentional and unintentional TUTs were: (a) Unintentional TUT rates dropped more from the SART to flanker task than did intentional TUT rates, and; (b) No-go response accuracy was lower preceding unintentional than intentional TUT reports.

Should intentional and unintentional TUT rates be made with similar confidence? Should they correlate equivalently with executive ability? Should their rates differentially change between tasks? Should they be differentially sensitive to errors? If all our findings were reversed, would they make researchers any less (or more?) confident about the validity of intentionality reports? Here is the crux of the problem: We cannot say what findings support or refute the validity of intentionality reports, and we cannot say what findings support or refute theories of intentional or unintentional mind wandering. We may hold out hope that accumulating data will eventually contribute to stronger validation and theories of intentionality and mind wandering, but without any such theory to leverage from the outset, it is difficult to see whether the field is making—or can soon make—any progress.

### Limits on generalizability and future directions

With so few studies critically investigating the measurement of mind-wandering, or its differential measurement across various probe methods, there is still much we do not know. The study’s limitations suggest some priorities for future construct validation work.

#### Operationalization of mind wandering

Of broadest concern, our operationalization of mind wandering was limited to TUT, but others are possible (see Christoff et al., 2018, b; Seli, Kane, et al., 2018a, b). Probes for alternative definitions of mind wandering, such as context-independent thought, or unconstrained thought, might yield different results.

#### Probe types

Because our primary findings indicate at least some differences in TUT reporting from different probe types, the most obvious limitation to the present work is that it was based on only a few (albeit representative) probe types. Even within each probe type we investigated, other versions are possible: content probes could emphasize the temporal orientation of TUT contents (e.g., past-, present-, future-oriented thoughts), intentionality probes could ask about intentionality of on-task thoughts as well as TUTs, and depth probes could use 0–10, 0–100, or −5 to +5 response scales, or scales with different verbal labels for anchors and midpoints, any of which might exert some subtle effects on thought reports. Future construct validation research must strike a balance between replicating prior findings (e.g., Robison et al., 2019; Schubert et al., 2020; Weinstein et al., 2018) and generalizing results across additional probe types.

There are many additional probe types for future research to consider, but we would prioritize two. First, the perspective that mind-wandering should be defined as unconstrained, freely moving thought (e.g., Christoff et al., 2016, Christoff et al., 2018, b; Irving, 2016), has recently inspired thought probes to assess this dimension (Mills et al., 2018; see also Murray et al., 2020). We have concerns about thought probes that require subjects to (a) infer constraint, which may share the same vulnerabilities to bias and confabulation as intentionality reports, and (b) retrospect over non-specified durations in order to infer and report thought movement. Moving-thought probes might therefore be validated against think-aloud protocols, which yield a relatively continuous report of the contents of consciousness (see Sripada and Taxali, 2020),^8^ and against methods that infer thought movement by examining thought-content consistency across consecutive probes (e.g., Welhaf et al., 2020; Zanesco, 2021).

Second, recent neuroimaging studies (e.g., Ruby et al., 2013; Smallwood et al., 2016; Wang et al., 2018), have resurrected the practices of daily-life studies (Klinger, 1978–1979; Klinger & Cox, 1987–1988), where each probe occasion asks multiple questions about different dimensions of thought (e.g., on- versus off-task; self- versus other-oriented; past versus future temporal orientation). These multi-dimensional experience sampling (MDES) probes have the obvious benefit of collecting more information about each experience and doing so within subjects and occasions. Validation research will be necessary, however, to determine whether subjects’ responses to each question within MDES probes change systematically with the order of those questions in the sequence, as time passes from the conscious experience and previous ratings may influence or interfere with later ones.

#### Task and cultural contexts

We probed TUTs during only two tasks in a fixed order (confounding order with task)—SART and flanker tasks—both of which were computer-based, with simple and repetitive decision rules, and with minimally engaging items presented on isolated trials. Both tasks also tapped into mental processes related to executive control, and both may have elicited at least some error-related reactivity, where preceding performance errors may have influenced TUT reporting (for some subjects, some of the time). Although our measuring TUTs in two tasks allows more generalizability than would only one, the similarities and differences we found in TUT reporting to different probe types may be specific to the task types we employed and the order in which we employed them. Tasks that involve remembering or integrating information over time, or that draw on people’s prior knowledge or interests, or that involve complex motor sequences, or that engage emotion, or that change dynamically with practice or skill, might yield different results. Given the breadth of tasks and activities that are used in the mind-wandering literature, from computerized choice-RT tasks, to reading texts, to watching video lectures, to simulated automobile driving, it is especially important to explore the construct validity of probed TUT rates across a variety of contexts.

Our study design was also limited to presenting retrospective questionnaire measures after subjects had already completed two thought-probed tasks. Perhaps the failure to find measurement invariance for our questionnaire battery across probe types resulted from different probe-type experiences and responses changing the way subjects interpreted questionnaire items or recalled their general propensities. Any such reactivity could then also have influenced the patterns of correlations between the TUT rates and questionnaires across probe types. Future construct-validation work seeking to assess the nomological network around probed TUT rates should vary task and questionnaire order or separate these measurements over long enough periods to minimize reactivity in either direction.

Finally, like many studies in psychology, our subject sample was restricted to young-adult undergraduates in a single Western, educated, industrialized, rich, and democratic (“WEIRD”) cultural context (e.g., Arnett, 2008; Henrich et al., 2010; Rad et al., 2018). Although research on mind wandering from non-WEIRD contexts has indicated similar results to those from WEIRD contexts (e.g., Iijima & Tanno, 2012; Lu et al., 2015; Shukor, 2005; Song & Wang, 2012; Zhang & Kumada, 2017), there are few such studies and so cross-cultural generalization is an open empirical question. One might expect that mind-wandering research, which requires self-disclosure and may reflect some biases due to folk theoretical commitments, may be vulnerable to cross-cultural threats to construct validity. Particularly relevant to the present study, if cultures differ in their folk conceptions of intentionality, or in their interpretations of the ostensible continuum of on-task to off-task thinking, they might produce discrepant results from those presented here.

### A provisional endorsement of content-based thought probes and a plea for better measurement

While mindful of this study’s limitations, we make a cautious recommendation: *Unless contraindicated by their specific research question, laboratory investigations of TUT should favor content-based thought probes*. Our findings suggested that TUT reports elicited by content probes were more stable across tasks, were made with higher confidence (and with more similar confidence to on-task reports), and were less vulnerable to reactivity from performance errors than were intentionality and depth probes. Moreover, only content probes yielded significant correlations in both SART and flanker tasks between TUT rate and executive attention (although content-probe correlations weren’t significantly larger than the other probe types’). We speculate that content probes are less vulnerable to reactivity, confabulation, and bias than other probe types (including more generic “*on-task versus off-task*” probes) because they demand subjects to commit to a specific mind-wandering experience, such as thinking about a persistent worry, or an impending errand, or a heroic fantasy, rather than simply reporting an off-task thought of no particular kind. That is, it should be harder to speciously endorse a TUT report when a probe requires the specification of experience.

More generally, we ask mind-wandering researchers to consider the validation lessons from DES (e.g., Hurlburt, 2011) and take more seriously how demand characteristics from repeatedly probing for TUTs (or other dimensions of mind wandering) might influence subjects’ thought reports, and how iterative self-report practice with feedback might improve their validity (Hurlburt and Heavey, 2015). Open-ended thought probes are rarely used in mind wandering studies (Baird et al., 2011; Rummel et al., 2017), but they might prove valuable as control conditions against which thought reports from closed-ended probe types are compared. We call for more humility regarding the construct validity of probed TUT reports, and urge the field to invest at least as much effort into the measurement quality of current probing methods as it does into the conceptual and theoretical demand to probe for increasingly numerous and subtle dimensions of mind-wandering experiences (e.g., Mills et al., 2018; Murray et al., 2020; Ruby et al., 2013)

### Supplementary Information

ESM 1 (DOCX 237 kb)

## Appendix

We dropped all questionnaire data, but retained cognitive-task data, for subjects who did not complete the questionnaire battery. We excluded all data for subjects with missing data for the SART (performance or probe responses), antisaccade letters, or arrow flanker probe responses, but we retained data for five subjects missing only the antisaccade arrows task; we favored antisaccade letters over antisaccade arrows for inclusions because it came first and was not accidentally presented as differently across testing sites (see *Method*). All case-wise and task-wise exclusions are specified below and were determined according to the order of criteria presented below (e.g., if a subject’s data were excluded based on experimenter notes, they were not subsequently re-counted toward possible exclusion based on outlying task performance).

**Case-wise exclusions based on experimenter notes or missing tasks**

Following Kane et al. (2016), we planned to exclude all data from subjects who the experimenter noted to have fallen asleep during multiple tasks, but no one met this criterion. We excluded all data for 23 subjects who were missing performance data from the SART or antisaccade-letters, or who were missing probe data from either the SART or arrow flankers; these data were either truly missing (*n* = 16; mostly due to subjects leaving the session early), or they were “missing” because, following Kane et al., we excluded them due to the experimenter reporting that subjects fell asleep multiple times during a single task (*n* = 3) or that subjects did not follow task instructions (e.g., not continuing to respond to all task stimuli; *n* = 4).

We additionally dropped all data from 1 subject who reported that English was not their primary language, two subjects who reported having previously participated in a thought-probe study, one subject who was unable to use their right hand to perform the tasks, and one subject with a significant visual impairment. (These exclusion criteria were not anticipated in our analysis plan.) Excluding data from these 28 of the original 1108 subjects left data from 1080 subjects.

**Case-wise data exclusion of outlying scores**

We dropped all data for subjects with an outlying score on any executive-control performance measure (antisaccade-letters, antisaccade-arrows, SART d′, SART intraindividual RT SD). Following Kane et al. (2016), we defined outliers as any observations falling more than three times the interquartile range away from the upper or lower hinges of a boxplot. This criterion led to eliminating data from 13 subjects with outlying scores on SART intraindividual RT SD. We therefore retained performance data from 1067 subjects.

**Exclusions of all questionnaire data**

We retained task-performance data and thought-probe data from subjects for whom we had to eliminate questionnaire data, or who did not complete the questionnaires.

**Missing questionnaires**

Subjects tested during the first two semesters of data collection at WCU (*n* = 173) completed only the executive and probed tasks in a 60-min session. As well, we dropped all questionnaire data from one subject who did not complete the full battery.

**Infrequency, inconsistency, and skipped responses in questionnaires**

Of the 893 subjects with complete questionnaire data, we dropped data from 38 subjects for excessive Inconsistency scores, and then from 30 subjects with excessive Infrequency scores (see *Methods* for these criteria). Although unanticipated, we also dropped questionnaire data from three subjects who skipped more than two items (these subjects skipped enough items—8, 16, and 29 items—to make us suspicious of their data quality). After these exclusions, we retained and analyzed questionnaire data for 822 subjects.

## Footnotes

1 Note that some studies using unpredictable thought probes ask subjects to report on experiences over an extended time period, such as the interval since the last probe, which may be minutes in duration (e.g., Antrobus, 1968; Antrobus et al., 1966; Farley et al., 2013; Filler & Giambra, 1973; Giambra, 1989, 1995; Kucyi & Davis, 2014; Shaw & Giambra, 1993). These reports likely suffer from similar forgetting and mental-aggregation biases as do retrospective questionnaire measures (although perhaps of smaller magnitude), and so we will not consider them further.

2 Although Hutchison et al. (2020) also appear to have detected performance reactivity in their pupillometry study, the findings are not straightforward. Their Study 1 cued subjects on each trial to expect a prosaccade trial (a target appearing in the same location as a cue) or an antisaccade trial (a target appearing in the opposite location as a cue) 500–8000 ms later; probes appeared after target-identification responses. TUT rates were slightly higher following antisaccade than prosaccade trials, but pupillometry measures indicated smaller pupils (often associated with TUTs) before prosaccade than antisaccade trials. Given that TUT reports may have been reactive to frequent antisaccade errors, Study 2 replaced some targets with probes, thus eliminating reactivity. Here, TUT rates were lower for antisaccade than prosaccade trials and pupils showed similar patterns as Study 1, and so Study 1 TUT rates may have been contaminated by reactivity. However, if antisaccade errors artificially increased TUT reports, then Study 2 should have reduced TUT rates; in fact, TUT rates on antisaccade trials *increased* slightly, from .41 in Study 1 to .43 in Study 2 (proaccade TUT rates increased from .38 to .48). It is unclear, then, whether the Hutchison et al. (2020) findings suggest error reactivity.

3 The finer-grained questions asked (and analyses reported) here were not included in our original analysis plan

4 We could not conduct LMMs in R as in our prior analyses (where data were organized into rows for each thought probe), because the probes created an unbalanced design: TUTs had two levels (intentional versus unintentional) but on-task responses had one. For all intentionality analyses, then, we used simplified datafiles with one row per subject, consisting of intentional TUT, unintentional TUT, and for some analyses, on-task thought report rates or means, calculated across all probe trials

5 Was et al. (2019) addressed a similar issue in a video-lecture study, by probing thoughts *related* to the lecture content but not focused on the here-and-now of it (“task-related” TUT; TRT). In Study 1, probes presented a TRI option among an on-task option and various TUT options, whereas in Study 2, probes additionally presented a TRT option. Mean TRT rate was .17. Although TRI rates were relatively stable across experiments (.19 vs. .16), on-task reports dropped in the presence of the TRT option (from .24 to .19), as did TUTs (from .57 to .48). The cross-experiment differences suggest that, when TRT is not a response option—at least for studies using content-laden tasks—subjects sometimes will characterize them as on-task thoughts and sometimes as TUTs.

6 Cheyne et al. (2009) may provide a starting point for this measurement pursuit, as their model of attention lapses proposed different types of performance errors to result from different gradations (or severities) of attentional withdrawal from the SART

7 We are unaware of straightforward methods to implement BFs for statistical comparisons of correlation coefficients, which might aid interpretation of the non-significant differences between correlations we frequently found

8 Mills et al. (2018) did something close to this, analyzing data from 18 subjects who responded to thought probes while engaged in 30 min of silent thought. At each probe, subjects rated whether their mind had been moving freely on a 1–7 scale and they typed a brief description of their thoughts since the last probe, in chronological sequence. A blind rater used the thought descriptions to rate how freely moving each thought sequence was, and in a linear mixed effects regression the subjects’ and blind-rater’s ratings correlated significantly (*p* = .046). We hope to see larger replications of this finding but note some concerns. First, if the theory is that mind-wandering should be defined as unconstrained, *freely* moving thought—and not just moving thought—and if subjects are told to rate whether their thoughts were “freely moving” and not just “moving,” we cannot rely on varied thought content as evidence for unconstrained thought; indeed, varied thought content may arise from subjects more frequently reorienting to on-task thinking in a controlled manner after catching themselves mind-wandering (Welhaf et al., 2020). Second, subjects’ ratings of more versus less freely moving thought in the moment may have biased their recollection and written expression of their prior thought content, artificially driving their ratings closer to those of the blind rater. Having subjects think aloud throughout the intervals and having them report more qualitatively about how they judge free movement of thought might provide more compelling evidence for the validity of unconstrained-thought reports
