# Supplementary material for: Testing the construct validity of competing measurement approaches to probed mind-wandering reports
Source: Behav Res Methods. 2021 Apr 9;53(6):2372–411. doi: 10.3758/s13428-021-01557-x (PMC8613094; doi:10.3758/s13428-021-01557-x)
Supplement: Supplementary file 1 — (DOCX 237 kb) [file 13428_2021_1557_MOESM1_ESM.docx]

**SUPPLEMENTAL MATERIALS**

Supplemental Table 1A. Correlation matrix for task-unrelated thought (TUT) rate and questionnaire measures in the total sample.

|  |
| --- |

**Note**. Bolded italicized correlations: ≥.30 across all 5 correlation matrices.

| Supplemental Table 1B. Correlation matrix for task-unrelated thought (TUT) rate and questionnaire measures in the content-probe condition.   |
| --- |

Supplemental Table 1C. Correlation matrix for task-unrelated thought (TUT) rate and questionnaire measures in the intentionality-probe condition.

|  |
| --- |

| Supplemental Table 1D. Correlation matrix for task-unrelated thought (TUT) rate and questionnaire measures in the depth-probe condition.   |
| --- |

Supplemental Table 1E. Correlation matrix for task-unrelated thought (TUT) rate and questionnaire measures in the content-probe condition without a task-related interference (TRI) response option.

|  |
| --- |

***Supplemental Table 2.*** Details about the questionnaire factor analyses.

Exploratory factor analyses (with promax rotation) for each probe condition separately yielded two factors with Eigenvalues >1 and two factors above the scree (see Supplemental Table 3); re-running each analysis to force a two-factor solution yielded results presented in Supplemental Table 4. As in the correlations, there is *some* consistency among probe-type conditions: One factor (“*Apathetic Inattention*”) was primarily loaded by measures of conscientiousness (negatively), boredom proneness, inattentiveness, and mind wandering; another factor (“*Positive Daydreaming But Pressured Thought*”) was primarily loaded by daydreaming frequency, problem-solving and purposeful daydreaming, openness, and racing and unwanted thoughts. However, these thematically central measures varied in their relative loadings (and some cross-loadings) across probe conditions, and additional measures varied in whether they loaded moderately or not on these factors.

Because our goal was to simplify our questionnaire measures into a few interpretable constructs (rather than to discover some “true” latent structure for the entire battery), we attempted to maximize the chance of finding factor invariance across probe-type conditions by conducting a multigroup CFA on only a subset of measures with relatively consistent loadings across groups: The “*Apathetic Inattention*” factor was indicated by 8 measures (ADHD–Hyperactivity, ADHD–Inattentiveness, CFQ–MAL, Conscientiousness, Fidgeting–SAQ, IPI–Boredom Proneness, IPI–Mind Wandering, and Mind Wandering–Spontaneous) and the “*Positive Daydreaming But Pressured Thought*” factor was indicated by 9 measures (IPI–Daydream Frequency, IPI–Mentation Rate, IPI–Problem-Solving Daydreams, Mind Wandering–Deliberate, Mind Wandering–Spontaneous, Openness, Metacognitive Prospective Memory, Schizotypy–Magical Ideation; White Bear Suppression Inventory). Perhaps unsurprisingly, given the exploratory factor analysis results, the multigroup CFA failed to indicate factor invariance (see Supplemental Table 5): The baseline, configural model did not adequately fit the data for all groups, indicated by a significant chi-square test, χ^2^(408) = 1843.81, *p* < .001, and constraining factor loadings to be equal across groups (“weak invariance”) significantly hurt model fit versus the configural model.

| Supplemental Table 3. Scree plots from exploratory factor analyses (promax rotation) of the questionnaire battery, by probe-type condition.  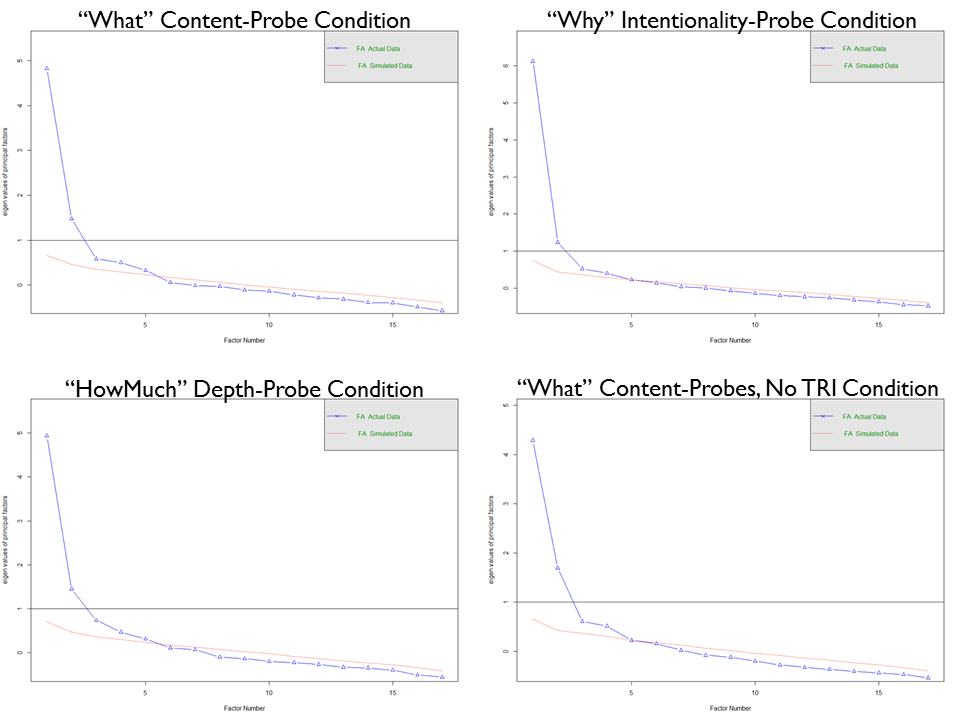 |
| --- |

Supplemental Table 4. Factor loadings from exploratory factor analyses (promax rotation) of the questionnaire battery, by probe-type condition.

|  | “What” Probes | “What” Probes | “Why” Probes | “Why” Probes | “How Much” Probes | “How Much” Probes | “What” Probes (no TRI) | “What Probes (no TRI) |
| --- | --- | --- | --- | --- | --- | --- | --- | --- |
| Measure | Factor 1 | Factor 2 | Factor 1 | Factor 2 | Factor 1 | Factor 2 | Factor 1 | Factor 2 |
| ADHD-Hyperactive |  | .323 |  | **.530** | .223 | .316 | **.533** |  |
| ADHD-Inattentive |  | **.603** |  | **.766** |  | **.566** | **.761** |  |
| CreativeAchieve | .235 |  | .221 |  | .330 |  |  | .323 |
| CFQ-MAL | .301 | **.501** | .269 | **.586** | .377 | .376 | **.658** |  |
| Conscientiousness |  | **−.780** |  | **−.699** |  | **−.781** | **−.630** |  |
| Fidget-SAQ | .289 | .297 | .294 | .459 | .330 | .261 | .437 |  |
| IPI-Boredom |  | **.718** |  | **.716** |  | **.650** | **.730** | −.258 |
| IPI-Daydream | **.745** | .212 | **.801** |  | **.812** |  | .293 | **.689** |
| IPI-MentationRate | **.550** | −.221 | **.607** |  | **.668** |  |  | .468 |
| IPI-MindWandering | .377 | **.530** | .352 | **.514** | .480 | .459 | **.637** | .217 |
| IPI-ProblemSolving | **.601** |  | **.791** |  | **.653** | −.280 |  | **.635** |
| MW-Deliberate | **.577** |  | **.723** |  | **.666** |  |  | **.609** |
| MW-Spontaneous | **.658** | .329 | **.621** | .369 | **.672** | .248 | **.548** | .443 |
| Openness | **.522** |  | **.585** |  | **.573** |  |  | **.645** |
| Metacog Prospective | **.562** | −.447 | **.508** |  | **.518** | −.456 | −.234 | .385 |
| Schiz-MagicIdeation | .334 |  | .354 |  | .339 |  |  | .237 |
| WhiteBear Suppress | **.621** |  | **.519** | .324 | **.544** |  | .353 | .267 |

**Note**. Factor loadings ≥.200 shown; factor loadings ≥.500 bolded. CreativeAchieve = Creative Achievement Questionnaire; CFQ–MAL = Cognitive Failures Questionnaire–Memory and Attention Lapses; Fidget–SAQ = Spontaneous Activity Questionnaire; IPI = Imaginal Process Inventory; MW = Mind Wandering; Metacog Prospective = Metacognitive Prospective Memory Battery; Schiz–MagicIdeation = Schizotypy–Magical Ideation Scale; WhiteBear Suppress = White Bear Suppression Inventory.

Supplemental Table 5. Test for measurement invariance in questionnaire measures with multigroup confirmatory factor analysis.

| Models | Df | AIC | BIC | χ^2^ | χ^2^ diff | Df diff | *p* |
| --- | --- | --- | --- | --- | --- | --- | --- |
| Configural Model | 408 | 21301 | 22243 | 1843.8 |  |  |  |
| Constrain Loadings | 453 | 21274 | 22005 | 1907.5 | 63.69 | 45 | 0.035 |
| Constrain Intercepts | 495 | 21241 | 21773 | 1957.8 | 50.32 | 42 | 0.177 |
| Constrain Means | 501 | 21234 | 21738 | 1963.0 | 5.22 | 6 | 0.516 |
